# Exogenous interleukin-1 beta stimulation regulates equine tenocyte function and gene expression in three-dimensional culture which can be rescued by pharmacological inhibition of interleukin 1 receptor, but not nuclear factor kappa B, signaling

**DOI:** 10.1007/s11010-023-04779-z

**Published:** 2023-06-14

**Authors:** Ross Eric Beaumont, Emily Josephine Smith, Lexin Zhou, Neil Marr, Chavaunne T. Thorpe, Deborah Jane Guest

**Affiliations:** 1 Clinical Sciences and Service, Royal Veterinary College, Hawkshead Lane, North Mymms, Hatfield, AL9 7TA Herts UK; 2 Comparative Biomedical Sciences, Royal Veterinary College, Royal College Street, London, NW1 0TU UK

**Keywords:** Tendon, Inflammation, Equine, Horse, Cytokine, Musculoskeletal

## Abstract

We investigated how Interleukin 1 beta (IL-1β) impacts equine tenocyte function and global gene expression in vitro and determined if these effects could be rescued by pharmacologically inhibiting nuclear factor-κB (NF-_K_B) or interleukin 1 signalling. Equine superficial digital flexor tenocytes were cultured in three-dimensional (3D) collagen gels and stimulated with IL-1β for two-weeks, with gel contraction and interleukin 6 (IL6) measured throughout and transcriptomic analysis performed at day 14. The impact of three NF-_K_B inhibitors on gel contraction and IL6 secretion were measured in 3D culture, with NF-_K_B-P65 nuclear translocation by immunofluorescence and gene expression by qPCR measured in two-dimensional (2D) monolayer culture. In addition, daily 3D gel contraction and transcriptomic analysis was performed on interleukin 1 receptor antagonist-treated 3D gels at day 14. IL-1β increased NF-_K_B-P65 nuclear translocation in 2D culture and IL6 secretion in 3D culture, but reduced daily tenocyte 3D gel contraction and impacted > 2500 genes at day 14, with enrichment for NF-_K_B signaling. Administering direct pharmacological inhibitors of NF-_K_B did reduce NF-_K_B-P65 nuclear translocation, but had no effect on 3D gel contraction or IL6 secretion in the presence of IL-1β. However, IL1Ra restored 3D gel contraction and partially rescued global gene expression. Tenocyte 3D gel contraction and gene expression is adversely impacted by IL-1β which can only be rescued by blockade of interleukin 1 receptor, but not NF-_K_B, signalling.

**Supplementary Information:**

The online version contains supplementary material available at 10.1007/s11010-023-04779-z.

## Introduction

Tendons are dense connective tissue primarily composed of a collagen I, proteoglycan and elastin extracellular matrix (ECM) with a majority tenocyte cell population [1, 2]. Tenocytes aid in the transduction of contractile forces from skeletal muscle to bone [1] and regulate ECM homeostasis and repair following tissue damage [3]. Tendon injuries account for 30% of all musculoskeletal injuries in humans [4] and 46% of limb injuries in racehorses [5]. Natural regeneration of the native tissue is poor and characterised by excessive collagen III deposition [6] resulting in biomechanically inferior scar tissue formation several months post-injury [7]. The disorganised fibrovascular matrix and impaired structural integrity predisposes horses to reinjury rates of up to 67% [8]. The human Achilles and equine superficial digital flexor tendon (SDFT) are frequently injured and share several risk factors including training and age [9, 10]. They both function as energy-storing tendons to facilitate locomotion [11] and equine tenocytes capture key molecular and cellular characteristics of human tenocytes in vitro, particularly collagen and ECM remodelling gene expression [12]. Consequently, the horse represents a valid large animal model to study human tendon injuries [13].

The mechanisms underlying tendon degeneration were suggested to be independent of inflammation [14]. However, the advent of cutting-edge molecular techniques supports its fundamental role in the aetiology of tendon disorders [7, 15–17]. While inflammation is necessary to facilitate tendon healing through the controlled regulation of pro-resolving mechanisms [7, 18], chronic inflammation is suggested to drive aberrant ECM remodelling and fibrosis [19, 20], with proinflammatory cytokines playing a key role in this process [21, 22]. Tenocytes secrete a variety of proinflammatory cytokines [23] and previous immunohistochemical studies detected interleukin 1 beta (IL-1β), tumour necrosis factor alpha (TNF-α) and interferon gamma (IFN-γ) in equine SDFT tissue obtained from injured Thoroughbred horses [24]. However, a recent in vivo time-course analysis of cytokine concentrations measured from equine SDFT ultrafiltrate revealed that only IL-1β and interleukin 6 (IL6) were elevated in the acutes stages following injury [25]. We previously demonstrated that stimulating equine tenocytes with IL6 does not negatively impact gene expression or three-dimensional (3D) collagen gel contraction in vitro [26], but exogenous IL-1β increases matrix metalloproteinase (MMP) gene expression in two-dimensional (2D) culture and impairs equine adult tenocyte 3D collagen gel contraction [27]. In the acutely injured tendon, aligned collagen is negatively correlated with the expression profiles of *MMP1, 3,* and* 9* [28]. Additionally, IL-1β enhances *MMP1* expression [27, 29, 30] which amplifies degradation of the damaged ECM [31], further weakening the structural integrity of the tissue. Similarly, transient IL-1β stimulation (24 h) modulates transcriptomic responses of rat Achilles’ tenocytes cultured in a 3D collagen gel [29] and increases cytokine and chemokine expression in human tenocytes [23, 32]. These observations suggest that IL-1β is a principal cytokine elevated during the early stages of tendon healing which elicits profound changes in tenocyte function and gene expression. However, no study has determined how exogenous IL-1β stimulation impacts global gene expression profiles in equine adult tenocytes following a stimulation protocol which resembles temporal inflammatory signalling in vivo [19, 25].

The tendon has a slow cellular and metabolic rate [33] resulting in prolonged recovery periods to establish normal functional capacity [34]. Current treatments include non-steroidal anti-inflammatory drugs (NSAIDs) to attenuate pain and swelling, but the global attenuation of inflammation can inhibit tissue healing by reducing cell migration and proliferation [35]. Additional options include platelet rich plasma (PRP) [36], gene therapies [37], biological scaffolds [38], and mesenchymal stromal cells (MSCs) [39]. Both PRP [40] and MSCs [39] are utilised to treat equine tendon injuries, with varying degrees of long-term success [41, 42]. Attenuating IL-1β signalling with the interleukin 1 receptor antagonist (IL1Ra) protein reduces inflammation in vivo both in humans [43] and horses [44]. Similar observations were reported in vitro, with ILR1a rescuing the impact of IL-1β on adult tenocyte 3D collagen gel contraction [27]. Additionally, IL1Ra reduced inflammation in a cartilage-synovium co-culture system [45] and modulated histopathological changes in an established tendinopathic rat model [46]. While clinical use of IL1Ra poses limitations including frequent dosing and a short biological half-life [43], the impact of IL1Ra on downstream tenocyte global gene expression profiles is unknown. Identifying changes in such genes and the signalling pathways in which they participate could elucidate the mechanism(s) through which IL1Ra and other IL1 inhibitors protect endogenous tenocytes from the deleterious effects of IL-1β.

Downstream of the IL1 receptor, IL-1β can activate different intracellular signalling pathways including c-Jun N-terminal Kinase (JNK) [47], p38 mitogen-activated protein kinase (p38-MAPK) [48], and extracellular-signal regulated kinase (ERK) [49]. However, emerging data suggests nuclear factor kappa-light-chain-enhancer of activated B cells (NF-_K_B) as a primary target of IL-1β [23, 27]. Following transient IL-1β stimulation, the NF-_K_B protein P65 translocates to the nucleus [27] to initiate gene transcription [26, 50]. Accordingly, small molecule inhibition of P65 attenuates NF-_K_B activation in murine tendon [51]. Upstream of the P65 protein is the iκb kinase (IKK) complex, comprising of the catalytic subunit IKKβ [50]. In mice, cre-mediated overexpression of IKKβ induced degeneration of the rotator cuff and enhanced cytokine concentrations in the joint, while IKKβ conditional knockdown improved tendon healing following an exercise overload model [23]. Furthermore, pharmacologically inhibiting IL-1β/IKKβ signalling in vitro reduced proinflammatory gene expression in human tenocytes [23]. Another kinase involved in IL1 signalling is interleukin-1 receptor-associated kinase 4 (IRAK4), with recent evidence from human clinical trials demonstrating that pharmacological inhibition of this kinase reduces inflammatory protein secretion by human stromal cells exposed to IL-1β [52]. Importantly, these in vitro studies of NF-_K_B inhibition were performed in 2D culture, which is a less efficient model to recapitulate the native tendon environment than a 3D culture model [53]. Similar 3D systems were utilised to examine therapeutics to alleviate tissue fibrosis [54] as they enable greater sensitivity than 2D monolayer systems by enabling cell-ECM interactions important for disease pathogenesis in vivo [55, 56].

The aim of this study was to investigate how exogenous IL-1β stimulation impacts equine tenocyte global gene expression utilising a physiologically relevant 3D culture system and determine if pharmacological inhibitors of IL1/NF-_K_B signalling can rescue the deleterious effects of IL-β. As IL-1B is elevated post-tendon injury [25] and activates NF-_K_B which drives tissue degeneration [23], identifying IL1/NF-_K_B inhibitors capable of restoring tenocyte function could serve as viable therapeutic strategies to protect endogenous tenocytes from excessive inflammation in vivo. In addition, changes in transcriptome signatures impacted by IL1/NF-_K_B inhibition may help elucidate downstream target genes which could also serve as therapeutic avenues to alleviate inflammation in the acute stages following a tendon injury.

## Materials and methods

### Tenocyte cell culture

Primary equine adult tenocytes (passage 3–8) derived from thirteen donors were utilised for all experiments. Cells were isolated post-mortem from Thoroughbred horses (aged between 2 and 17 years) that were euthanised for reasons unrelated to the project with the approval from the Royal Veterinary College Clinical Research Ethical Review Board (URN 2020 2017-2). As described [27], tissue samples were digested overnight at 37 °C and 5% CO_2_ in type 1 collagenase (1 mg/ml; Sigma-Aldrich, Dorset, UK). The isolated cells were expanded in complete media containing high glucose (4.5 g/l) Dulbecco’s modified eagle medium (DMEM) supplemented with 10% fetal bovine serum, 2 mM L-glutamine, 100 U/ml penicillin, and 100 µg/ml streptomycin (all Fisher Scientific, Hemel Hempstead, UK). When cells reached 80% confluency, they were passaged using 0.25% trypsin–EDTA (Sigma-Aldrich). Culture conditions were maintained at 37 °C and 5% CO_2_. An overview of the different tenocyte culture conditions is shown in Table 1 and described in subsequent sections.

**Table 1:** Tenocyte culture conditions and cytokine/drug stimulation for each experimental protocol

Treatment	2D culture	3D culture
IL1B (nM)	1^1,3,4^	1^2,5,6,7^
JSH23 (µM)	1^1,3,4^, 10^4^, 100^4^	1^2,5^, 5^2^, 10^2^, 25^2^, 50^2^
IMD0354 (nM)	100^1,3,4^, 500^4^, 1000^4^	100^2,5^, 1000^2,5^
PF-06650833 (nM)	100^1,3,4^, 500^4^, 1000^4^	100^2,5^, 1000^2,5^
IL1Ra (ng/ml)	N/A	100^2,7^

72 h stimulation^1^; Collagen gel contraction^2^; Immunofluorescence^3^; Presto blue cell viability^4^; IL6 secretion^5^; Cytokine array^6^; RNA sequencing^7^; N/A = not applicable. Note: Vehicles used were either water, PBS, or culture media at 1:100–1:1000 concentration

### Two-dimensional tenocyte stimulation

For 2D stimulation experiments, tenocytes were seeded at 3 × 10^4^ cells/cm^2^ and 24 h later exposed to IL-1β (1 nM; Peprotech, London, UK) in the presence and absence of the NF-κB inhibitors JSH23 (1 μM; Abcam, Cambridge, UK), IMD0354 (100 nM; Abcam) or PF-06650833 (100 nM, Cambridge Bioscience, Cambridge, UK) for 72 h. Media without IL-1β/NF-κB inhibitors served as the unstimulated control. Three biological replicates were used per condition for these experiments (passage 4–8).

### Three-dimensional (3D) tenocyte culture

3D culture was performed as described [27]; briefly, silicone-coated 6-well plates (Dow Corning Sylgard 184 Silicone elastomer; Farnell, Leeds, UK) were fitted with pairs of 0.2-mm-diameter minutien pins (Interfocus fine science tools, Cambridge, UK) manually embedded at 15 mm distances. Tenocytes were suspended (4 × 10^5^ cells/ml) in a chilled mixture of eight parts PureCol (Bovine collagen type I; Advanced Biomatrix, Carlsbad, CA, USA) and two parts complete media (pH adjusted from 7.2 to 7.6 with 1 M sodium hydroxide); 200 μl of collagen-cell suspension was pipetted around each pair of pins and the parafilm-sealed plates were left to set at 37 °C for 60–90 min. Subsequently, 3 ml of complete media was added to each well, supplemented with IL-1β (1 nM) and/or IL1Ra (100 ng/ml; Sigma-Aldrich) with fresh media changes every 3–4 days for 14 days. The NF-κB inhibitors JSH23 (1–50 μM), IMD0354 (100–1000 nM) or PF-06650833 (100–1000 nM) were also added to the media with and without the presence of IL-1β (1 nM). Media without cytokines/inhibitors served as the unstimulated control and all media changes occurred every 3–4 days. For the NF-κB inhibitor experiments, day 14 cell survival was determined by digesting 1–3 constructs in 1 mg/ml of type I collagenase (Sigma-Aldrich) for 1–2 h at 37 °C with results displayed as a percentage of cells seeded relative to day 0. For all 3D culture experiments, ImageJ software (National Institutes of Health) was used to measure contraction rates over time, with values displayed as the percentage size relative to day 0. Three lines of tenocytes were used in these experiments, except for JSH23 with two biological replicates per condition (all passage 3–7).

### RNA isolation and extraction

RNA samples from both 2D and 3D culture were extracted with Tri-reagent (Sigma) and purified using an RNAeasy mini kit (Qiagen, Manchester, UK) with contaminating genomic DNA removed with Ambion DNA-free (Life Technologies, Paisley, UK). For 3D culture samples, RNA concentrations were measured using a Qubit (ThermoFisher, Loughborough, UK) and purity (260/280 ratio > 1.8) was determined using a DS-11 spectrophotometer (DeNovix, Wilmington, US) with RNA integrity confirmed (> 1.8) on a Tapestation (Agilent, Milton Keynes, UK). For 2D culture samples, RNA purity (260/280 ratio > 1.8) and concentrations were measured using a DS-11 spectrophotometer (DeNovix).

### RNA sequencing

Eight lines of adult tenocytes (passage 3–7) were used in these experiments. Oxford Genomics (Oxford, UK) and Novagene (Cambridge, UK) isolated and prepared the mRNA fraction from total RNA and converted to end-paired cDNA with adapter ligation. Libraries were size selected, multiplexed, and quality controlled before pair-end sequencing (150 bp) over one unit of a flow cell on a NovaSeq6000 platform, generating 30.7–46.2 million reads per sample. The FASTQ files underwent quality control using FASTQC (version 0.11.9; Babraham Bioinformatics, Cambridge, UK). Trimming/filtering of reads was not required due to the high sequence quality and minimal adapter content. Reads were aligned to the *Equus caballus* transcriptome (EquCab3.0 GCF_002863925.1) using the pseudoaligner Salmon [57] (version 1.8) in Quasi-mapping-based mode with GC-bias correction (-gcBias). Tximport [58] (version 1.24) was used to import quantified gene level abundance data into R (version 4.2.1) and differential expression analysis was performed using R/Bioconductor DESeq2 [59] (version 1.36). Genes with an adjusted *p* value of < 0.05 and a log2-fold change (Log2FC) of ± 1 were considered differentially expressed (DE). The biological processes predicted to be affected by the differentially expressed genes were identified using the Gene Ontology (GO) software Panther GO-slim (www.pantherdb.org) and GO terms with a False Discovery Rate (FDR) of < 0.05 were considered significantly enriched. Pathway analysis was performed with Enrichr (Enrichr (maayanlab.cloud)) utilising the BioPlanet 2019 resource and terms with an adjusted *p* value of < 0.05 were considered significantly enriched. For both GO and pathway analysis, DE genes were analysed separately as up-and-down regulated lists, respectively. When too few DE genes were present to elicit any significant output from Panther GO-slim and Enrichr, STRING (STRING: functional protein association networks (string-db.org)) was utilised to examine functional interaction networks with the *Equus caballus* (9796) reference.

### cDNA synthesis and qPCR

cDNA was generated from 500 to 1000 ng of input RNA utilising the sensiFAST cDNA kit (Bioline, London, UK). To ensure no genomic DNA was present, reactions lacking the reverse transcriptase (-RT) enzyme were performed. Primers were designed with NCBI Primer-Blast (https://www.ncbi.nlm.nih.gov/tools/primer-blast/) to obtain amplicons with a size of 50–150 bp and melting temperatures of 59–61 °C. Primer sequences (Sigma, UK) are shown in Table 2. For each qPCR reaction, 10 ng of cDNA or -RT were added to SYBR Green containing supermix (Bioline) in duplicate 96-wells and analysed on a CFX96 C1000 thermal cycler (Bio-Rad, United states). Reactions lacking cDNA (-RT controls) were used to ensure no genomic DNA amplification or contamination of the SYBR mastermix was present. Cycling parameters were 95 °C for 10 min, followed by 45 cycles of 95 °C for 15 s, 60 °C for 15 s and 72 °C for 15 s. Subsequently, a melt curve was produced with readings every 1 °C from 65 to 95 °C. Gene expression levels were quantified relative to the housekeeping gene 18 s RNA using the 2^−ΔΔCt^ method [60] and data was expressed as a fold change relative to the untreated control.

**Table 2:** Primer sequences used for qPCR analysis

Gene	Protein name	Forward primer	Reverse primer
*18S rRNA*	18 s ribosomal RNA	CCCAGTGAGAATGCCCTCTA	TGGCTGAGCAAGGTGTTATG
*FGF19*	Fibroblast growth factor 19	GTGGAGATCAGAGCAGTCGC	CTCCTCGAAGGCGCAGTC
*IL-1β*	Interleukin 1 beta	CTCCTCGAAGGCGCAGTC	CCACAAGACAGGTACAGGTTCT
*CSF3*	Colony Stimulating Factor 3	TGAGGAAGATCCAGGCCGATG	AGAGTGTCCCAGCAGCATGAG
*SCX*	Basic helix-loop-helix transcription factor scleraxis	CCCAAACAGATCTGCACCTT	ATCCGCCTCTAACTCCGAAT
*TNC*	Tenascin	AACCCGTCCAAAGAGACCTT	GCGTGGGATGGAAGTATCAT
*MKX*	Homeobox protein Mohawk	AAGGCAAAGGAACCATTCGG	TTAGCTGTCACCCTTATTGGAT
*COL5A1*	Collagen Type V Alpha 1 Chain	AGGAGAGAGAGGCCCAAAAG	CTCCATCAATTCCCTGAGGA
*COMP*	Cartilage oligomeric matrix protein	AGAACATCATCTGGGCCAAC	CGCTGGATCTCGTAGTCCTC
*SOX9*	Transcription factor SOX-9	GCTCTGGAGACTTCTGAACGA	GTAATCCGGGTGGTCCTTCT
*MMP1*	Matrix Metallopeptidase 1 (Interstitial collagenase)	CTTTGATGGACCTGGAGGAA	GAATGGCCAAATTCATGAGC
*MMP3*	Matrix Metallopeptidase 3 (Stromelysin-1)	TGGACCTGGAAAAGTTTTGG	GACCAAGTTCATGAGCAGCA
*MMP8*	Matrix Metallopeptidase 8 (Neutrophil collagenase)	TTTGATGGACCCAATGGAAT	TTCATGGGCAGCAACAATAA
*MMP9*	Matrix Metallopeptidase 9	GAGATCGGGAATCATCTCCA	CCAAGAGTCGCCAGTACCTC
*MMP13*	Matrix Metallopeptidase 13 (Collagenase 3)	GCCACTTTGTGCTTCCTGAT	CGCATTTGTCTGGTGTTTTG

### Immunofluorescence on 2D coverslips

Adult tenocytes (*n* = 3, passage 4–8) were seeded on gelatin (Sigma)-coated coverslips in 24-well plates and stimulated 24 h later with IL-1β (1 nM) for 60 min with or without the NF-κB inhibitors JSH23 (1 μM), IMD0354 (100 nM), or PF-06650833 (100 nM). Complete media without IL-1β or the NF-κB inhibitors served as the unstimulated control. Subsequently, cells were fixed in 3.7% paraformaldehyde for 20 min and permeabilised for 1 h at room temperature with 0.1% triton-X-100/phosphate buffered saline (PBS). Blocking was performed with 2.5% normal horse serum (Vector Laboratories, Peterborough, UK) for 20 min at room temperature. Incubation with the mouse anti NF-κB-p65 primary antibody (Table 3) was carried out in blocking solution overnight at 4 °C. The secondary antibody (1:200; goat anti-mouse IgG Alexaflor 594™, Thermo Fisher) and phalloidin (1:500; Abcam) incubation was performed in blocking buffer for 3 h at room temperature. A secondary antibody-only staining served as the negative control. Coverslips were mounted with Vectashield Hardset containing DAPI (4′,6-diamidino-2-phenylindole, Vector Laboratories). Nuclear staining intensity was quantified by measuring the mean grey scale of the nucleus with ImageJ.

### Confocal imaging analysis of 3D constructs

Tenocyte 3D constructs (*n* = 2, passage 6–7) were prepared for confocal imaging as described [61], with minor modifications. Constructs were incubated with the primary antibody mouse anti-Collagen Type I (Table 3) for 80 h at 37 °C. Subsequently, an anti-mouse secondary antibody (1:200; goat anti-mouse IgG Alexaflor™ 594, Thermo Fisher) and phalloidin (1:500) were incubated for 36 h at 37 °C before an overnight incubation with DAPI (0.5 µg/ml, Fisher Scientific). Prior to tissue clearing, constructs were washed, dehydrated with methanol and cleared with HISTO™-M. Constructs were imaged as described [62]. Constructs were immersed in HISTO™-M on a glass-bottom dish for imaging. Serial optical sections (z-step size: 7.5 µm) of each construct was acquired using a Leica TCS SP8 laser scanning confocal microscope equipped with a motorised stage. Images were acquired with a HC PL FLUOTAR 10×/0.32 dry objective lens, with resolution set to 1024 × 1024 px, a pinhole size of 1 Airy unit, a frame average of 1 and a line average of 1. Tile scans of tendons were captured using light-emitting lasers at 405 (blue channel; DAPI), 488 (green channel; Phalloidin), and 561 (red channel; Alexa Fluor® 594) nm to detect fluorescent signal, with scanning speed set to 600 Hz. All settings, including laser power, were identical for imaging of all constructs. 3D renderings were obtained using Leica LAS X software (version 3.5.5) within the 3D module.

**Table 3:** Primary antibodies used for immunofluorescence

Marker	Species	Dilution	Company
NF-κB (p65)	Mouse	1:100	Thermo Fisher (436,700)
Collagen 1 alpha 1	Mouse	1:100	Novus biologicals (NB600-450)

### Presto blue cell viability assay

Cell viability was determined with the PrestoBlue™ reagent (Thermo Fisher) assay. Tenocytes were seeded in 96-well plates at 1.5 × 10^4^ cells/well in complete media. After 24 h, fresh complete media was added containing IL-1β (1 nM) in the presence and absence of JSH23 (1, 10, and 100 μM), IMD0354 (100, 500 and 1000 nM) or PF-06650833 (100, 500, and 1000 nM). Stimulations were 72 h and media without IL-1β or the NF-κB inhibitors served as the unstimulated control. After incubation, media was removed and 100 μl of diluted (1:10) PrestoBlue™ reagent was added to each well and left to incubate for 30 min at 37 °C. Fluorescence was measured at an excitation wavelength of 560 nm and an emission wavelength of 590 nm on a Tecan plate reader (Infinite M Plex; Tecan, Switzerland) utilising three biological replicates per condition (passage 4–8).

### Interleukin 6 ELISA and cytokine antibody array

IL6 was measured from the conditioned media of adult tenocyte 3D gels (*n* = 4, passage 4–7) treated with IL-1β (1 nM) or control (0 nM) for 14 days, with sample collection on days 4, 7, 10, and 14, respectively. Additionally, IL6 was determined at these time points from the conditioned media of 3D gels exposed to the NF-κB inhibitors JSH23 (1 μM), IMD0354 (100 and 1000 nM), and PF-06650833 (100 and 1000 nM) in the presence and absence of IL-1β (1 nM) with two (JSH23) or three (IMD0354 and PF-06650833) biological replicates per condition (passage 3–7). Collected media were briefly centrifuged at room temperature for 2 min at 10,000 xg, with the remaining supernatant immediately stored at −70 °C until analysis. IL6 was determined with an equine IL6 ELISA kit (R&D systems, US) according to the manufacturer’s instructions. Colorimetric detection was performed on a Tecan plate reader (Infinite M Plex) at 450 nm, with background correction at 540 nm. An eight-point standard curve with four-parameter logistic regression was utilised to calculate absolute IL6 concentrations (pg/ml).

Day 14 conditioned media samples from IL-1β (1 nM) and control (0 nM) 3D culture samples were also analysed for the presence of IL15, IL2, IL4, IL8, MCP-1, VEGFα, IFN-γ, IL-1α, IL1Ra, and IL10 with an equine cytokine array (RayBiotech, US) according to the manufacturer’s instructions. Membranes were imaged on a GBOX ChemiDoc (Syngene, India) with signal intensity quantified with ImageJ and protein levels expressed as fold change relative to the unstimulated control.

### Statistical analysis

All statistical analysis was performed with SPSS (version 28.0; IBM, UK). An independent t-test was utilised to compare two means. When comparing more than two means, a one-way ANOVA with Tukey post-hoc was employed. Normality was checked with the Shapiro–Wilk test and the equality of variance was determined with Levene’s test of homogeneity. If these assumptions were violated, data was log-transformed prior to analysis. If this did not result in a normal distribution of the data the non-parametric Kruskal–Wallis test was performed followed by Dunn’s pairwise comparisons with a Bonferroni correction. Variables measured over time were analysed with a one-way repeated factor ANOVA. If the assumption of sphericity was violated, a greenhouse–geisser correction was applied. When two or more groups were analysed over time, a two-way mixed factor ANOVA with a Tukey or Bonferroni post hoc correction for multiple comparisons was employed. In all cases *p* < 0.05 was considered statistically significant.

## Results

### IL-1β stimulation elicits robust changes in global gene expression patterns and collagen gel remodelling by adult tenocytes after 14 days in 3D culture

**Fig. 1:**
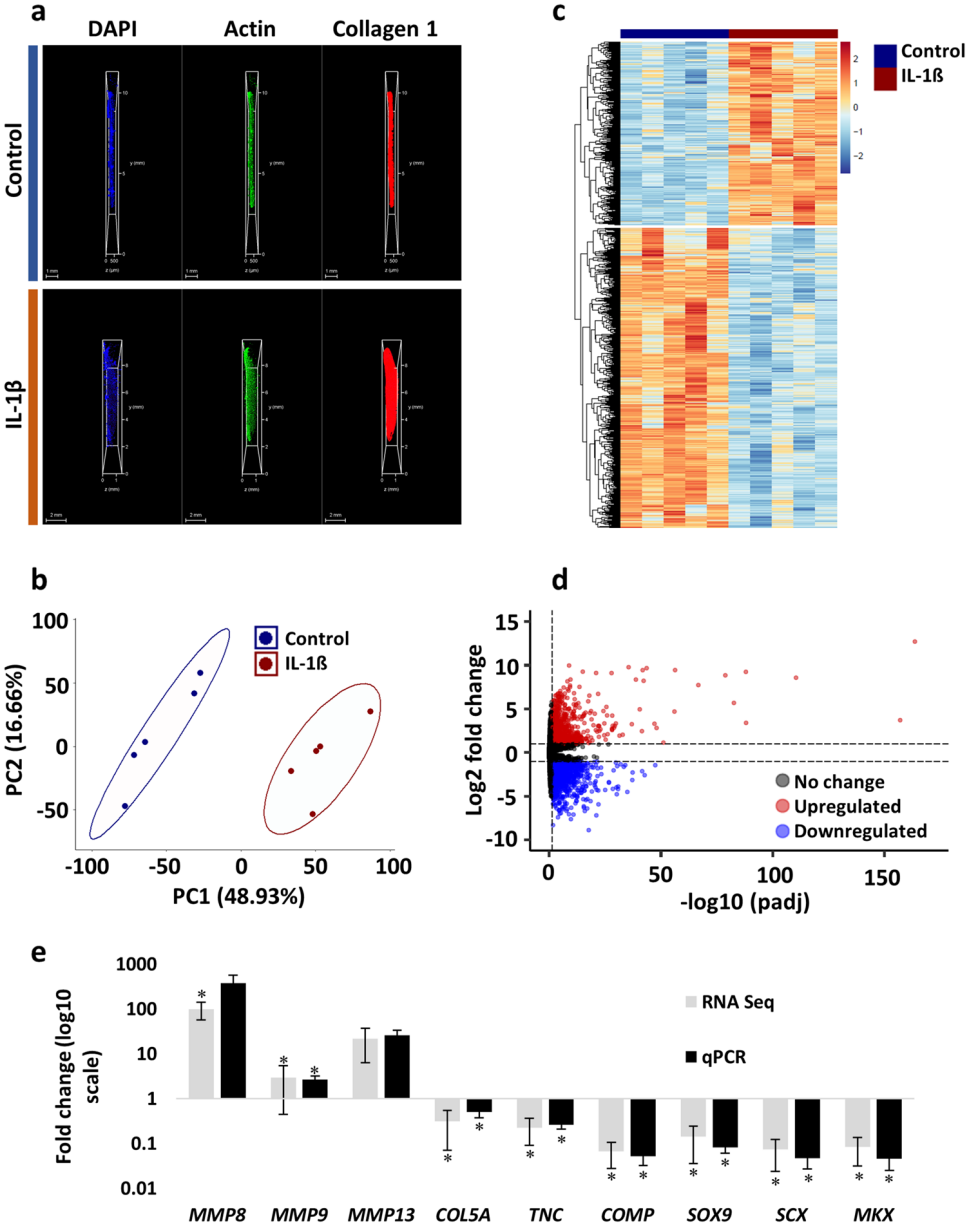
Morphological and transcriptomic responses of equine adult tenocytes exposed to IL-1β for 14 days in 3D culture. **A** Whole-mount confocal images of the tendon constructs at day 14; collagen 1 is shown in red, actin filaments in green and nuclear staining by DAPI is shown in blue. Scale bars = 1 mm for the control and 2 mm for the IL-1β samples. Images are representative of *n* = 2. **B** PCA of global gene expression profiles from five biological replicates in control (blue) and IL-1β (red) conditions. **C** Heatmap depicting the 2517 DE genes (adjusted *p* value < 0.05 and log2-fold change ± 1). Samples are visualised in columns (control blue, IL1-β red) and genes are represented and clustered (euclidean) by row. **D** Volcano plot highlighting the 18,435 genes in the final dataset; red (*n* = 954) and blue (1563) dots represent the significantly up-and-down regulated genes, respectively. The X-axis represents the -log 10 (adjusted *p* value) and the y-axis depicts the log2-fold change. **E** In-house qPCR validation of nine genes impacted by IL-1β. Values are mean ± SEM (*n* = 5) of fold change versus the unstimulated control. *denotes *p* < 0.05 versus control determined by the Wald test (RNA Seq) or an Independent t-test (qPCR)

As described [27], IL-1β reduces 3D collagen gel contraction by equine adult tenocytes and modulates tendon-associated and extracellular matrix remodelling gene expression in 2D culture. Therefore, we harvested 3D constructs at day 14 to characterise morphological and transcriptomic responses to IL-1β stimulation. Whole-mount confocal microscopy revealed differences in gel size (Fig. 1A), with the IL-1β constructs approximately two-fold wider than control by day 14 (Online Resource 1 and 2). Transcriptomic analysis revealed clear segregation of the five control and five IL-1β samples by principal component analysis (PCA; Fig. 1B). Of the 18,435 filtered genes, quantitative analysis (adjusted *p* value < 0.05 and log2-fold change ± 1) yielded 2517 DE genes impacted by IL-1β (Fig. 1C); of these, 954 were upregulated (Online Resource 3) and 1563 were downregulated (Online Resource 4), respectively (Fig. 1D), with selected genes showing good agreement with in-house qPCR analysis (Fig. 1E). A panel of ten cytokines and chemokines were analysed from the conditioned media of 3D gels at day 14. Of these, only IL8, MCP1, and VEGFα were detected across both conditions (Fig. S1A) and only IL8 was upregulated following IL-1β stimulation (Fig. S1B). The cytokines *IL2* and *IL4* had zero counts mapped to their respective genes across both conditions (Online Resource 5) and only IL8 was significantly upregulated at both the mRNA (Fig. S1C) and secreted protein level (Fig. S1B) following IL-1β stimulation.

### Enrichment analysis reveals NF-_KB_ as the primary inflammatory pathway activated by IL-1β in adult equine and murine tenocytes in 3D culture

**Fig. 2:**
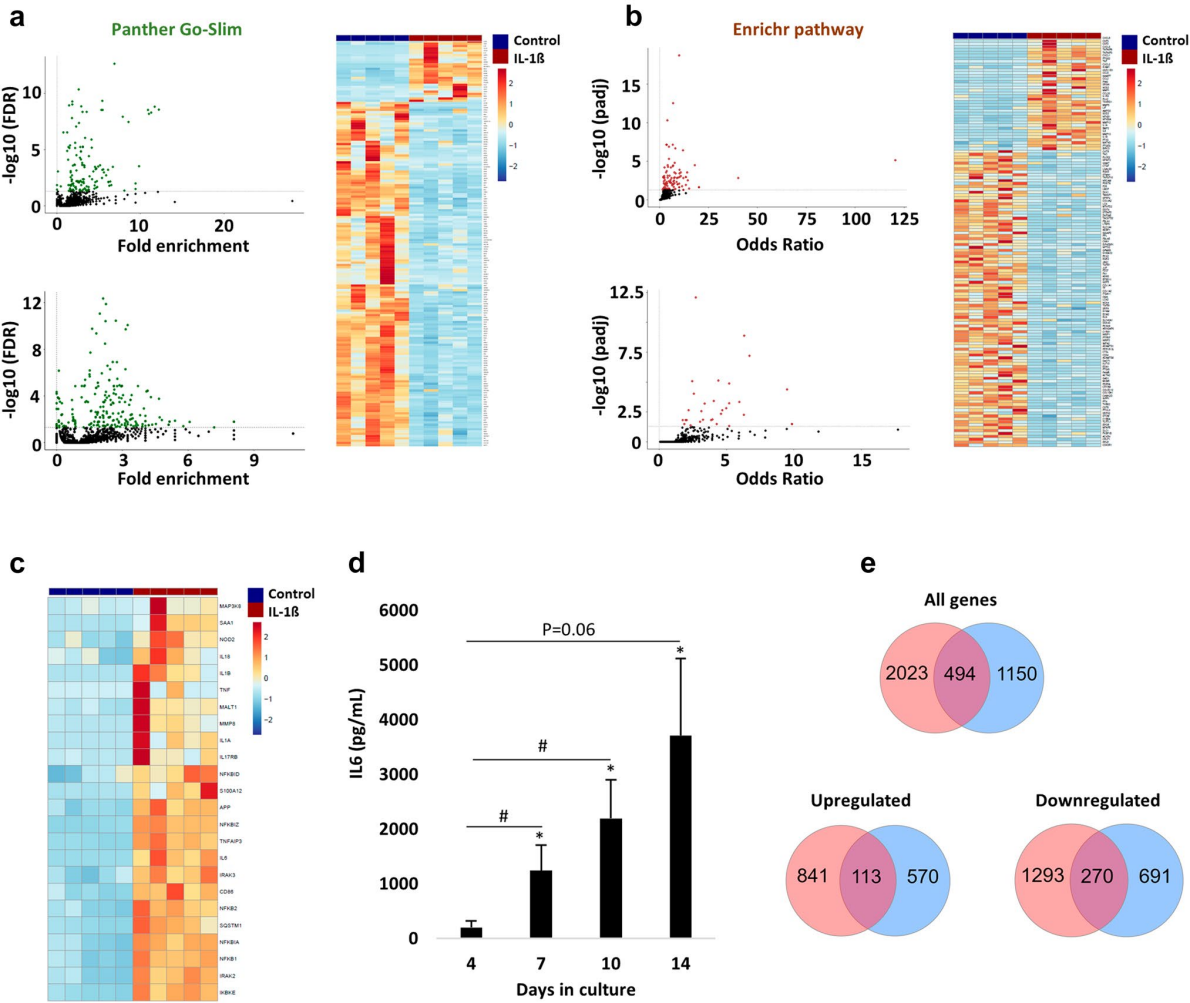
Enrichment analysis, IL6 secretion, and overlap of DE genes impacted by IL-1β in 3D culture.** A** Scatter plots highlighting the GO biological processes terms. Significant terms (FDR < 0.05) are shown in green for the upregulated (upper, *n* = 159 hits) and downregulated (lower, *n* = 198 hits) DE genes. The heatmap shows the DE genes (normalised counts) mapping to ‘Inflammatory response’ (top cluster) and ‘Anatomical structure development’ (bottom cluster); samples are visualised by column (control in blue, IL-1β in red) and genes are visualised and clustered (euclidean) by row. **B** Scatter plots highlighting the Enrichr pathway analysis. Significant terms (padj < 0.05) are shown in red for the upregulated (upper, *n* = 104 hits) and downregulated (lower, *n* = 31 hits) DE genes. The heatmap shows the DE genes (normalised counts) mapping to ‘Interleukin-1 regulation of ECM’ (top cluster) and ‘TGF-beta regulation of ECM’ (bottom cluster); samples are visualised by column (control in blue, IL-1β in red) and genes are visualised and clustered (euclidean) by row. **C** Heatmap depicting various NF-_K_B regulatory and target genes (normalised counts) significantly upregulated by IL-1β. Samples are visualised by column and genes are visualised and clustered (euclidean) by row. **D** Secretion of IL6 measured from the conditioned media of 3D constructs throughout the 14-day culture period. No IL6 was detected in the control samples. *and # denotes *p* < 0.05 versus control (i.e., 0) and day 4 (IL-1β condition), respectively, determined by Bonferroni post hoc following a significant (*p* < 0.05) two-way ANOVA. Values are mean ± SEM (*n* = 4). **E** Overlap of DE genes with Gehwolf et al. [29] after applying the same statistical filtering (adjusted *p* value < 0.05 and log2-fold change ± 1). Red (our data), blue [29]

GO and Enrichr pathway analysis was performed on the DE genes impacted by IL-1β (Fig. 2A and B). Heatmaps were constructed with the normalised counts for the upregulated and downregulated DE genes present in the top enrichment terms for GO (Fig. 2A, right image) and Enrichr (Fig. 2B, right image). The top hits for the GO were ‘inflammatory response’ (upregulated; Fig. S2A) and ‘anatomical structure development’ (downregulated; Fig. S2B). Furthermore, the top hits for Enrichr were ‘Interleukin 1 regulation of the extracellular matrix’ (upregulated; Fig. S2C) and ‘TGF-beta regulation of the extracellular matrix’ (downregulated; Fig. S2D). We next determined which specific inflammatory pathway was enriched in the GO and Enrichr analysis of the 954 upregulated DE genes. The proinflammatory JNK, P38-MAPK, ERK and STAT3/5 pathways were not enriched in either analysis, but the NF-_K_B pathway was enriched in both the GO (Online Resource 6) and Enrichr (Online Resource 7) analysis. Accordingly, multiple NF-_K_B regulatory genes (*NFKB1, NFKB2, NFKBIZ, NFKBIA*) and direct targets (*IL6, TNFα, IL1α*) were upregulated by IL-1β (Fig. 2C). Furthermore, secreted IL6 was enhanced by IL-1β throughout the 14-day culture period, but was undetectable in control samples (Fig. 2D). Subsequently, we cross-refenced our transcriptomic results with a recent study [29] which utilised a rat Achilles’ tenocyte 3D model; of the 133 shared genes augmented by IL-1β (Fig. 2E), multiple NF-_K_B genes and target products (*NFKBIA, NFKBIZ, IRAK3, IL6, IL1α*) were present (Online Resource 8). Consistent with recent findings [23, 27], these analyses suggest that IL-1β elicits its proinflammatory effects by activating the NF-_K_B pathway.

### The NF-_KB_ P65 translocation inhibitor JSH23 confers little rescue to adult tenocytes in the presence of IL-1β

**Fig. 3:**
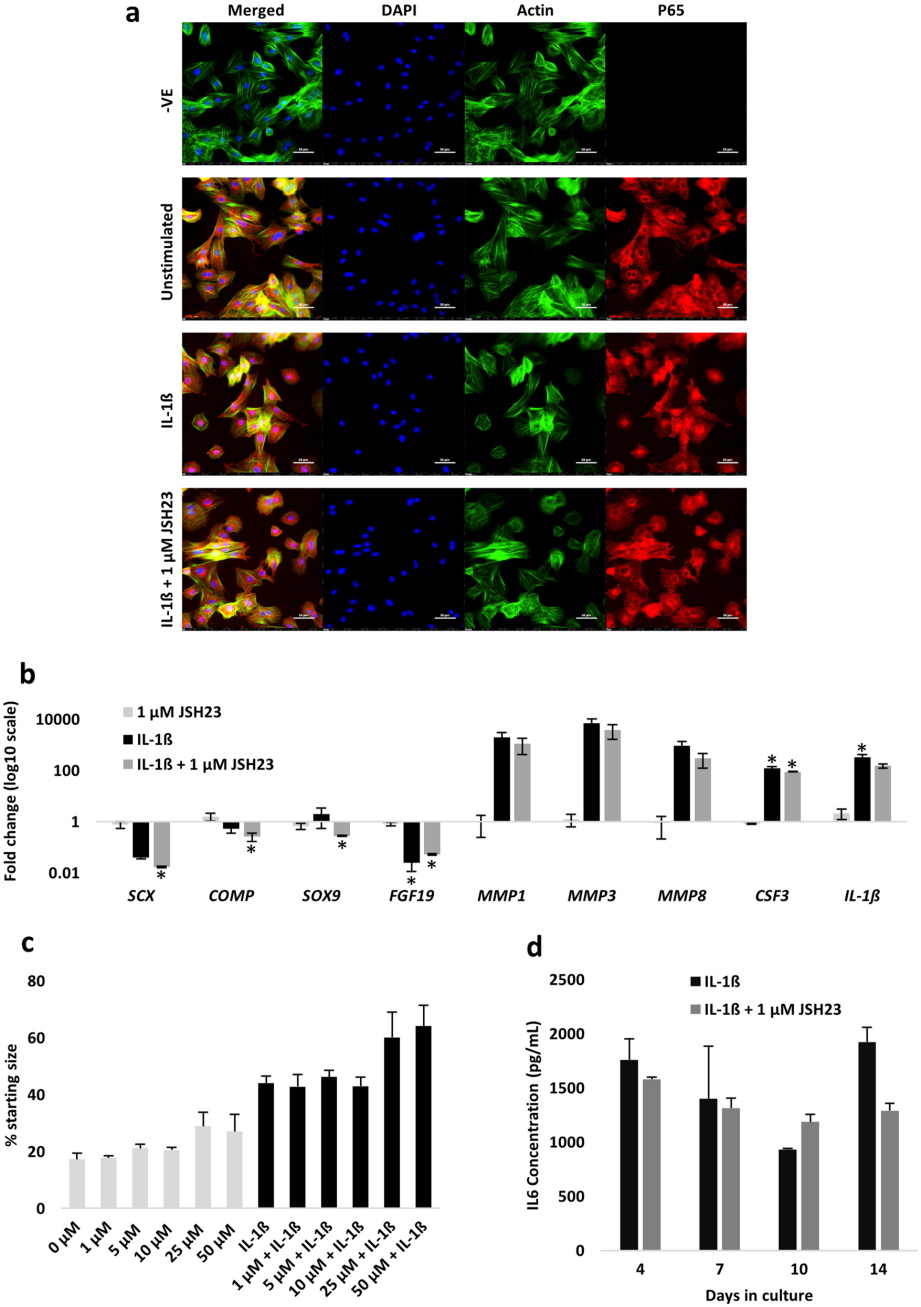
P65 inhibition by JSH23 does not restore tenocyte gene expression, collagen gel contraction or IL6 secretion in the presence of IL-1β. **A** 2D immunofluorescence staining of P65 cytosol/nuclear shuttling following 60 min of IL-1β stimulation (1 nM) with and without JSH23 (1 µM). Unstimulated cells served as the control. P65 is shown in red, actin filaments in green, and nucleus staining by DAPI in blue. Scale bar = 50 µM. Images are representative of three biological replicates. **B** Fold change in gene expression following 72 h stimulation with JSH23 (1 µM), IL-1β (1 nM), or both. Unstimulated cells served as the control (i.e., 1). *Denotes *p* < 0.05 versus control with Tukey or Dunn post hoc following a significant (*p* < 0.05) one-way or Kruskal–Wallis ANOVA. Values are mean ± SEM (*n* = 3). **C** Day 14 gel size expressed as a percentage relative to day 0. Concentrations of JSH23 ranged from 1–50 µM with (black bars) and without (grey bars) IL-1β (1 nM), respectively. Values are mean ± SEM (*n* = 2). **D** IL6 secretion over the 14-day 3D culture period with IL-1β (1 nM) or IL-1β (1 nM) + JSH23 (1 µM). No IL6 was detected in the JSH23 (1 µM)-only or control conditions. Values are mean ± SEM (*n* = 3)

As the NF-_K_B P65 protein rapidly shuttles from the cytosol to the nucleus following transient IL-1β exposure [27], we pharmacologically inhibited NF-_K_B signalling with the P65 translocation inhibitor JSH23. We selected a 1 µM dose for the initial immunofluorescence experiments as this was sufficient to inhibit P65 translocation when co-administered with IL-1β (Fig. 3A). However, this dose did not rescue IL-1β-induced changes in gene expression (Fig. 3B). Prior to performing 3D culture experiments, we tested higher doses of JSH23 (10 and 100 µM) and observed no adverse effect on cell viability after 72 h stimulation in 2D culture (Fig. S3A). Day 14 collagen gel size was examined following JSH23 concentrations ranging from 1 to 50 µM in the presence and absence of IL-1β; all doses failed to restore tenocyte gel contraction when co-administered with IL-1β (Fig. 3C). These impairments in 3D collagen gel contraction were not due to differences in cell survival at day 14, although there was a trend for a decrease in survival with the provision of 25–50 µM doses when administered with IL-1β (Fig. S3B). Finally, JSH23 (1 µM) did not attenuate IL-1β-induced increases in IL6 secretion over the 14-day culture period (Fig. 3D).

### Inhibition of IKKβ by IMD0354 does not rescue IL-1β-induced changes in gene expression, collagen gel contraction or IL6 secretion

**Fig. 4:**
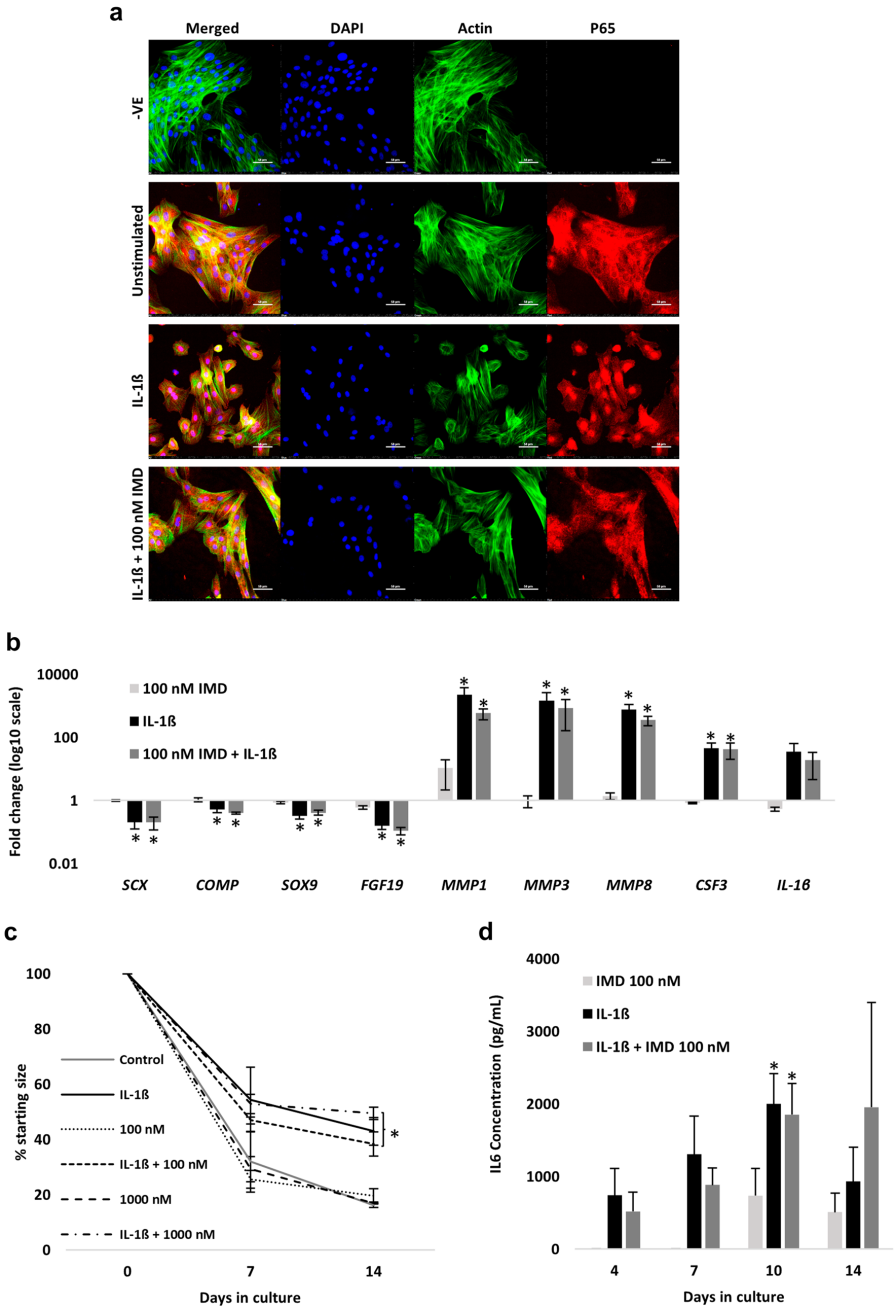
IKKβ inhibition by IMD0354 does not rescue the impact of IL-1β on tenocyte gene expression, collagen gel contraction or IL6 secretion. **A** 2D immunofluorescence staining of P65 cytosol/nuclear translocation following 60 min of IL-1β stimulation (1 nM) with and without IMD0354 (100 nM). Unstimulated cells served as the control. P65 is shown in red, actin filaments in green, and nucleus staining by DAPI in blue. Scale bar = 50 µM. Images representative of *n* = 3. **B** Fold change in gene expression following 72 h stimulation with IMD0354 (100 nM), IL-1β (1 nM), or both. Unstimulated cells served as the control (i.e., 1). *denotes *p* < 0.05 versus control with Tukey or Dunn post hoc following a significant (*p* < 0.05) one-way or Kruskal–Wallis ANOVA. Values are mean ± SEM (*n* = 3). **C** Changes in collagen gel size over the 14-day period expressed as a percentage relative to day 0. Concentrations of IMD0354 were 100 and 1000 nM, respectively, with and without IL-1β (1 nM). Values are mean ± SEM (*n* = 3). *denotes *p* < 0.05 versus control and IMD3054-only conditions following a significant (*p* < 0.05) two-way (time x condition) ANOVA. **D** IL6 secretion over the 14-day 3D culture period with IL-1β (1 nM) or IL-1β (1 nM) + IMD0354 (100 nM). No IL6 was detected in the control conditions. Values are mean ± SEM (*n* = 3). *denotes *p* < 0.05 versus control following a significant (*p* < 0.05) two-way (time x condition) ANOVA. IMD = IMD0354

Pharmacological inhibition of IKKβ attenuates inflammatory gene expression and IL6 secretion in human tenocytes stimulated with IL-1β in 2D culture [23]. Therefore, we exposed adult tenocytes to IL-1β with and without the IKKβ inhibitor IMD0354. A concentration of 100 nM had no impact on cell viability after 72 h in 2D culture (Fig. S4A) but reduced P65 nuclear translocation when co-administered with IL-1β for 60 min (Fig. 4A). However, IMD0354 did not rescue the impact of IL-1β on 2D gene expression after 72 h (Fig. 4B) or 3D gel contraction over two-weeks (Fig. 4C); the latter was not attributed to differences in cell survival at day 14 (Fig. S4B). Furthermore, IKKβ inhibition did not reduce IL-1β-induced increases in IL6 secretion in 3D culture (Fig. 4D).

### Inhibition of IRAK4 by PF-06650833 modestly attenuates gene expression in 2D culture, but does not rescue 3D gel contraction or IL6 secretion

**Fig. 5:**
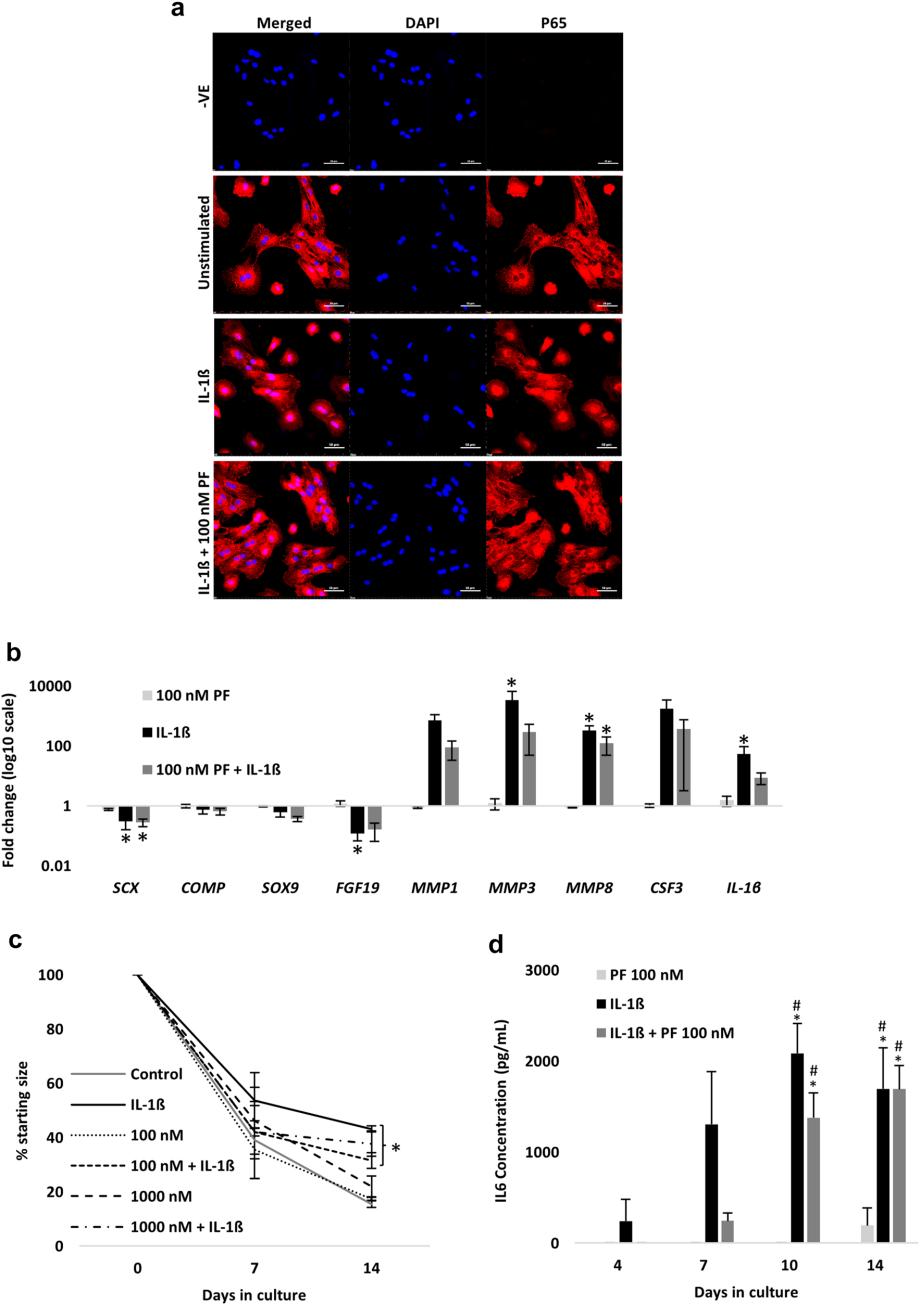
IRAK4 inhibition by PF-06650833 confers moderate rescue of tenocyte gene expression in the presence of IL-1β. **A** 2D immunofluorescence analysis of P65 cytosol/nuclear staining following 60 min of IL-1β stimulation (1 nM) with and without PF-06650833 (100 nM). Unstimulated cells served as the control. P65 is shown in red and nucleus staining by DAPI in blue. Scale bar = 50 µM. Images are representative of three biological replicates. **B** Fold change in gene expression following 72 h stimulation with PF-06650833 (100 nM), IL-1β (1 nM), or both. Unstimulated cells served as the control (i.e., 1). *denotes *p* < 0.05 versus control with Tukey or Dunn post hoc following a significant (*p* < 0.05) one-way or Kruskal–Wallis ANOVA. Values are mean ± SEM (*n* = 3). **C** Changes in collagen gel size over the 14-day period expressed as a percentage relative to day 0. Concentrations of PF-06650833 were 100 and 1000 nM, respectively, with and without IL-1β (1 nM). Values are mean ± SEM (*n* = 3). *denotes *p* < 0.05 versus control and PF-06650833-only conditions following a significant (*p* < 0.05) two-way (time x condition) ANOVA. **D** IL6 secretion over the 14-day 3D culture period with IL-1β (1 nM), PF-06650833 (100 nM), or IL-1β (1 nM) + PF-06650833 (100 nM). No IL6 was detected in the control condition. Values are mean ± SEM (*n* = 3). * and # denotes *p* < 0.05 versus control and PF-06650833 (100 nM), respectively, following a significant (*p* < 0.05) two-way (time x condition) ANOVA. PF = PF-06650833

Next, we tested PF-06650833, a selective and potent inhibitor of IRAK4, a downstream kinase of the IL1 receptor which mediates NF-_K_B signalling [50]. PF-06650833 administered at 100 nM attenuated inflammatory gene expression in human stromal cells exposed to IL-1β in 2D culture [52] and is well-tolerated over a wide range of doses in humans [63]. Concentrations of 100–1000 nM did not influence cell viability (Fig. S5A) and 100 nM blocked P65 nuclear translocation (Fig. 5A) and prevented significant IL-1β-induced changes in *FGF19, MMP3*, and *IL-1β* gene expression in 2D culture (Fig. 5B). However, 100 and 1000 nM had no impact on 3D collagen gel contraction (Fig. 5C); this was not due to differences in cell survival at day 14 (Fig. S5B). Lastly, 100 nM of PF-06650833 did not attenuate IL6 secretion in the presence of IL-1β (Fig. 5D).

### Modulation of the equine tenocyte transcriptome by IL1Ra

Given none of the NF-_K_B inhibitors tested could attenuate the effect of IL-1β in 3D culture (gel contraction, IL6 secretion), we utilised the IL1 inhibitor demonstrated to fully restore collagen gel contraction when co-administered with IL-1β: the IL1 receptor antagonist protein IL1Ra [27]. This protein also blocks IL-1β-induced P65 nuclear translocation in equine tenocytes cultured in 2D [27]. Therefore, we harvested 3D collagen gels after 14 days of IL-1β stimulation (1 nM) with and without the provision of IL1Ra (100 ng/ml) and examined global gene expression profiles. As IL1Ra administered in isolation to equine tenocytes cultured in 3D had little impact on gene expression (Fig. S6A), these samples were not sent for RNA Sequencing. This resulted in three conditions: (1) unstimulated (negative) control, (2) IL-1β (positive) control and, (3) IL-1β + IL1Ra.

**Fig. 6:**
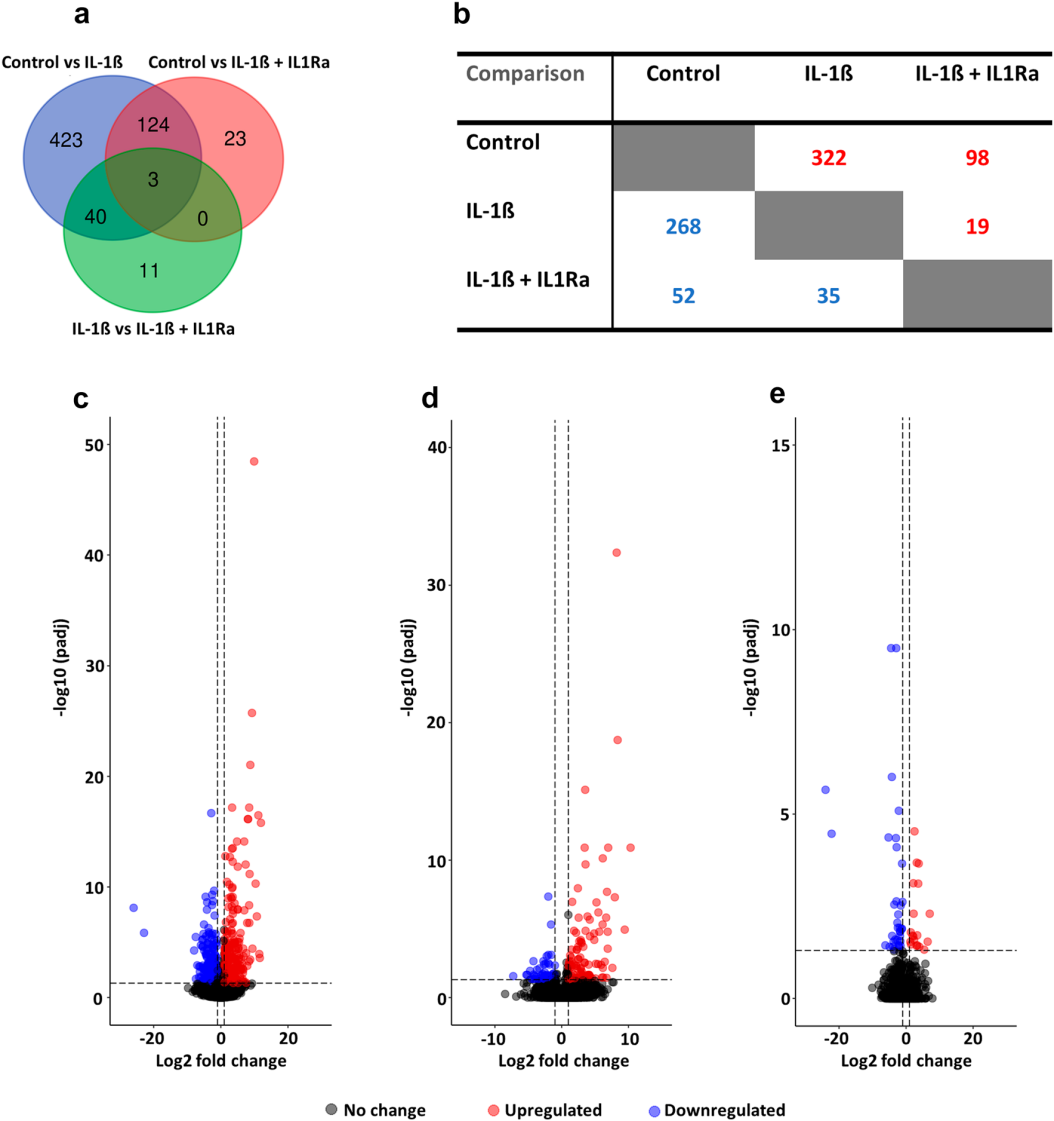
Magnitude of change in DE gene expression by IL1Ra after 14 days in 3D culture. **A** Venn diagram highlighting the common and unique DE genes for each pairwise comparison from the DESeq2 analysis. **B** Direction of change in DE gene expression for each of the three conditions; red and blue numbers denote up-and-down regulation of gene expression, respectively. **C–E** Volcano plots depicting the upregulated (red) and downregulated (blue) DE genes in the control vs IL-1β (**C**), control vs IL-1β + IL1Ra (**D**), and IL-1β vs IL-1β + IL1Ra (**E**) conditions, respectively. DE genes were determined based on a log2FC > 1 and padj < 0.05

The provision of IL1Ra fully restored tenocyte function in the presence of IL-1β, with similar contraction rates versus control (Fig. S6B). Additionally, day 14 transcriptomic analysis revealed that the IL-1β + IL1Ra treatment elicited fewer significant changes in gene expression (150) than IL-1β alone (590) versus control (Fig. 6A). Consistent with the first batch of global gene expression analysis, genes impacted by IL-1β included *MMP3*, *MMP8*, *MMP13*, *CSF3, IL6, IL-1β*, *TNC* and *SOX9*; the nucleotide metabolism genes *ENPP1, 2,* and* 5*; and the ECM gene *AB13BP*, respectively (Online Resource 9). The provision of IL1Ra did not rescue *MMP8*, *CSF3, IL6,* or *IL-1β* expression, but did attenuate *TNC*, *SOX9*, *ENPP1, 2, 5* and *AB13BP* (Online Resource 10). Furthermore, there were 54 DE genes in the IL-1β vs IL-1β + IL1Ra comparison (Online Resource 11). As the control and IL-1β + IL1Ra conditions resulted in similar contraction rates by day 14 (Fig. S6B), this suggests the 40 DE genes in the control vs IL-1β/IL-1β vs IL1β + IL1Ra overlay (Fig. 6A) could be involved in regulating tenocyte function. Accordingly, the eleven uniquely DE genes in the IL-1β vs IL-1β + IL1Ra comparison and the three genes DE across all treatments (*MIP-2BETA, GPR84* and *GLCCI1*; Fig. 6A) are unlikely to be mediating the functional rescue of IL1Ra on collagen gel contraction. Overall, stimulation with IL-1β resulted in more upregulated (322) than downregulated (268) genes relative to control (Fig. 6B). Similarly, when comparing IL-1β + IL1Ra vs control, more DE genes were increased (98) than decreased (52) in expression, respectively (Fig. 6B). Conversely, the co-administration of IL-1β + IL1Ra attenuated more DE genes (35) than upregulated (19) relative to IL-1β alone (Fig. 6B). To visualise the magnitude of changes in gene expression for each pairwise comparison, volcano plots were constructed to highlight the control vs IL-1β (Fig. 6C), control vs IL-1β + IL1Ra (Fig. 6D), and IL-1β vs IL-1β + IL1Ra (Fig. 6E) treatments, respectively.

### Network analysis indicates tenocyte collagen gel contraction is mediated by downstream regulation of nucleotide metabolism genes and ABI3BP

**Fig. 7:**
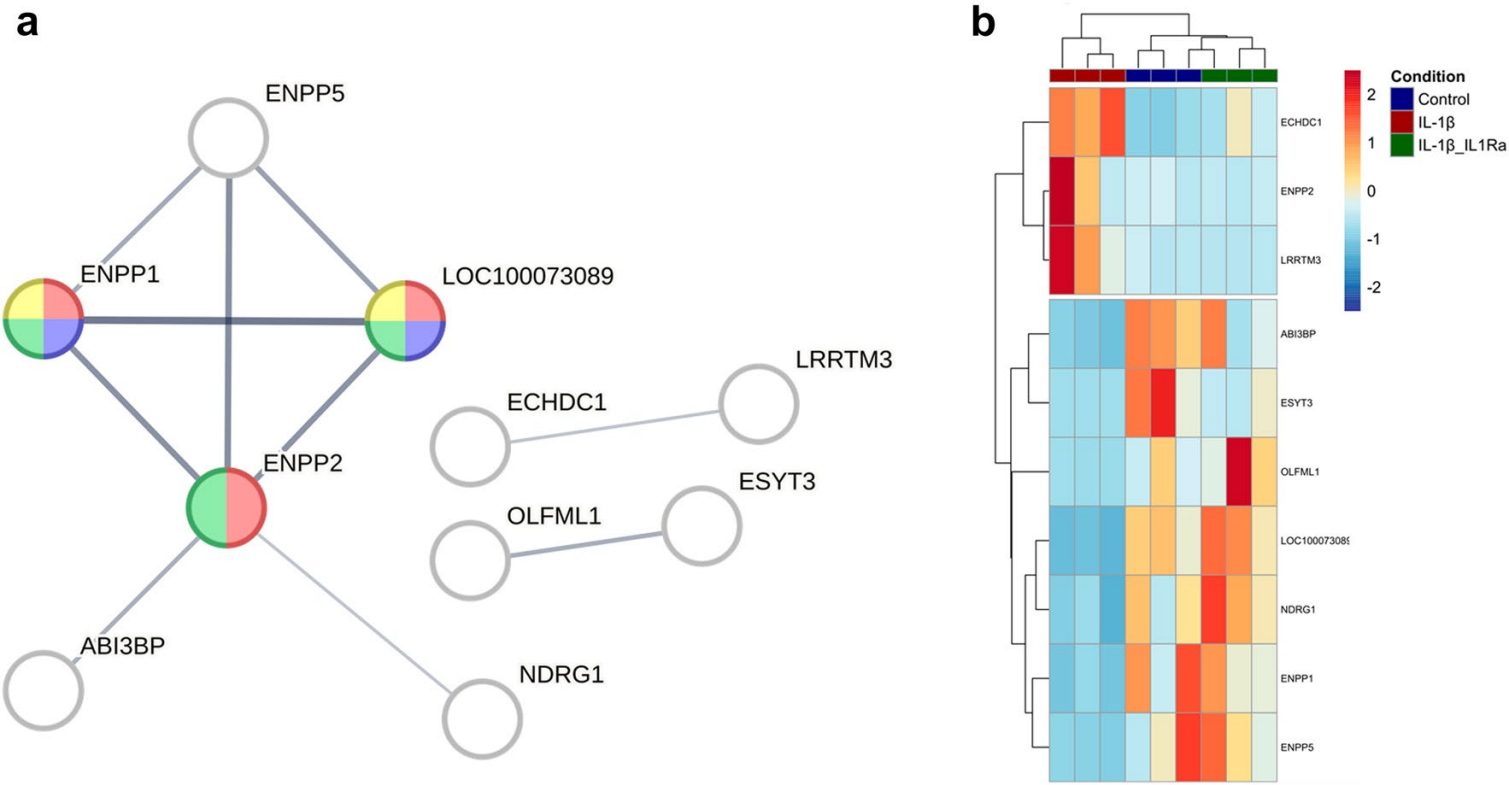
Network analysis and magnitude of change in expression of the DE genes suggested to regulate tenocyte collagen gel contraction. **A** Functional network interaction of the 33 DE genes conducted with STRING; only the proteins (nodes) with a predicted interaction score > 0.4 (medium confidence) are displayed (*n* = 10), with the remaining DE genes omitted. A thicker line (edges) indicates a stronger interaction score. GO enrichment analysis revealed significant hits (FDR < 0.05) in the molecular function category shown in red (GO:0004528), green (GO:0004551), and blue (GO:0036218), respectively. KEGG pathway indicated enrichment of one term (ecb00500) shown in yellow. **B** Heatmap depicting the normalised count data for the 10 DE genes isolated in the network analysis. Samples are visualised and clustered (euclidean) in columns (control blue, IL1-β red, IL-1β + IL1Ra green) and genes are represented and clustered (euclidean) by row

Consistent with the first batch of GO enrichment analysis, NF-_K_B was the only inflammatory pathway enriched in the control vs IL-1β comparison (Online Resource 12), which was no longer apparent in the presence of IL1Ra (Online Resource 13). The 40 DE genes in the control vs IL-1β/IL-1β + IL1Ra overlay did not elicit any significant results from the GO analysis with an FDR < 0.05. To further isolate the DE genes which may be responsible for mediating tenocyte 3D gel contraction, we cross-referenced these 40 DE genes with the first batch of control vs IL-1β analysis (Fig. 1) which delineated 33 genes DE across all eight biological replicates (Online Resource 14). These genes were then analysed with STRING to determine protein interaction networks (Fig. 7A). This resulted in ten significant interactions, with the ectonucleotides ENPP1, ENPP2, and ENPP5 exhibiting the strongest connections. The only ECM protein was ABI3BP, which displays independent connections with several proteins involved in collagen homeostasis (Fig. S7). The ten interactions were enriched (FDR < 0.05) for three molecular functions including ‘Phosphodiesterase I activity’ (red), ‘dTTP diphosphatase activity’ (blue) and ‘Nucleotide diphosphatase activity’ (green), respectively. The KEGG term ‘starch and sucrose metabolism pathway’ was also enriched (yellow; Fig. 7A). Three of the ten genes were upregulated by IL-1β and seven were downregulated, respectively (Fig. 7B).

## Discussion

Inflammation is a key driver of tendon degeneration [23] with elevated levels of IL-1β post-injury [25] suggested to mediate tissue breakdown [31] by regulating matrix remodelling and tendon-associated gene expression [27, 29, 32]. While transient IL-1β exposure (1–3 days) alters gene expression in 2D [27] and 3D [29] culture, no study has investigated how IL-1β impacts transcriptomic profiles and tenocyte function in 3D culture following a time-course which more closely resembles the acute inflammatory phase in vivo [19, 25]. Consequently, the current study investigated how exogenous IL-1β stimulation influences global gene expression and whether these changes can be modulated by pharmacological inhibition of IL1/NF-_K_B signalling.

Several reports highlight IL-1β-induced elevations in *MMP* gene expression by tenocytes isolated from equine [27] and murine [32] tissue. We confirm an upregulation of *MMP1, 2, 3, 8, 9* and *13*, but we did not measure MMP activity. Rat Achille’s tenocyte MMP2 and MMP9 activity was not impacted by IL-1β following pulsed electromagnetic treatment [29], although the IL-1β-treated cells were not directly compared to an unstimulated control. Elevations in MMP expression and activity following IL-1β stimulation may enhance degradation of the collagen-rich ECM in the acutely injured tendon [31] and our 3D culture system. However, simultaneous temporal profiling of collagen protein levels, MMP activity, and 3D gel contraction would need to be determined.

Additional genes were impacted by exogenous IL-1β stimulation, including an upregulation of the glycoproteins *CSF3* and *CSF2*. The protein encoded by *CSF3* mobilises leukocytes and stem cells from the bone marrow into the bloodstream in an IL-1β-dependent manner [64] and CSF2 acts locally to recruit tissue-resident macrophages [65]. While cellular infiltration can promote tendon healing, imbalanced influx can facilitate tissue degeneration [66] consistent with the observed differences in stromal and immune cell composition between human control and tendinopathic tissue samples [67]. Although we did not confirm *CSF3* and *CSF2* upregulation at the protein level, similar changes in expression were reported in rat Achille’s tenocytes exposed to IL-1β in a 3D collagen matrix [29]. The pleiotropic cytokine *IL6* was also elevated by IL-1β in the current study, consistent with other reports [29]. We recently showed that exogenous IL6 does not affect adult tenocyte gene expression or collagen contraction [26]. Alternatively, IL6 likely plays an immunoregulatory role in the tendon niche by coordinating progenitor/stem cell proliferation [68] and macrophage polarisation [69] during the proliferative phase of tendon healing [70], effects which would not be captured in our model.

Enrichment analysis of the DE genes revealed that NF-_K_B was the only proinflammatory pathway activated by IL-1β. Several studies demonstrate that NF-_K_B is activated during the acute stage of tendon healing in humans [23] and can persist into the remodelling phase in murine surgical models of tendon repair [19]. Upregulation of NF-_K_B signalling was also confirmed by our analysis of the DE genes reported by Gehwolf et al. [29], but several studies have shown that other pathways—including JNK [47], P38 [48] and ERK [49]—may also facilitate the proinflammatory effects of IL-1β. However, we demonstrated that key proteins of the JNK and P38 pathways do not translocate to the nucleus in equine adult tenocytes following transient IL-1β exposure [26]. Furthermore, IL-1β enhances ERK signalling in diseased, but not healthy, human tenocytes [49]. Whether ERK signalling would be enhanced by IL-1β in equine tenocytes isolated from tendinopathic SDFT tissue remains to be determined and could explain the lack of effect of IL-1β on ERK signalling in healthy tenocytes in the current study. Enrichment analysis also revealed that IL-1β attenuated TGFβ regulation of the ECM. Reducing TGFβ signalling could delay tendon healing as all three TGFβ isoforms help maintain ECM integrity through the deposition of collagens, fibronectin, and glycosaminoglycans [71] while also reducing IL-1β release from macrophages [72]. However, IL-1β increased the expression of the *TFGβ1* isoform in human tenocytes [49], yet we observed a slight reduction in *TGFβ1* expression (Log2FC = −1.99). Such discrepancies across studies may be explained by differences in cell culture models, IL-1β dosing strategies, and/or species effects.

While treatment with NSAIDs reduces inflammation, these drugs inhibit cell migration and proliferation [35] and may only confer benefit when administered during the remodelling phase of tendon healing [73]. The NF-_K_B pathway is activated during the early inflammatory stage of tendon healing [23] and IL-1β stimulation induces rapid nuclear translocation of the NF-_K_B protein P65 [27] with subsequent DNA binding [26] in adult tenocytes. Importantly, blocking P65 translocation with JSH23 attenuates inflammatory gene expression in rat tendons [51]. In the current study, JSH23 reduced IL-1β-induced P65 nuclear translocation but had little effect on gene expression in 2D culture; similar results were observed for the 3D measurements of collagen gel contraction and IL6 secretion. The reason for this is unclear, but the 1 µM dose of JSH23 is lower than utilised in other studies (30 µM) [51]. While higher doses of JSH23 may provide better rescue, we selected 1 µM as this concentration inhibited P65 nuclear translocation with no reduction in cell viability in 2D culture. Conversely, higher doses (25–50 µM) appeared to decrease 3D collagen gel contraction when co-administered with IL-1β with a corresponding decrease in tenocyte survival at day 14. However, we did not perform direct cell viability measurements throughout the 3D culture period, which could elucidate whether this reduction in gel contraction was due to a functional impairment of the tenocytes or an increase in apoptosis. Of note, Chen et al. [51] reported that JSH23 at 30 µM decreased proliferation and increased apoptosis in rat tenocytes.

In addition to JSH23, no rescue was provided by pharmacologically inhibiting IKKβ with IMD0354, conflicting with recent findings in humans [23] and rats [74]. In the study by Abraham et al. [23], healthy human tenocytes displayed reduced proinflammatory gene signatures following treatment with 1 ng/ml of IL-1β with and without 50 µM of an IKKβ inhibitor for 4 h. The lower IL-1β/IKKβ inhibitor ratio and/or stimulation timeframe relative to the present study may account for these divergent findings. A follow-up study by the same group tested a lower dose of an IKKβ inhibitor (1 µM) [74] which attenuated IL6 secretion in 2D culture and improved tendon healing in a rat rotator cuff model. However, in the current study, cell viability was reduced with 1 µM of IMD0354 with and without IL-1β; therefore, it is plausible that potential species variations in tenocyte responses [12] prevented higher concentrations of IMD0354 from being utilised without reducing cell viability. Furthermore, chronic IKKβ inhibition augments IL-1β secretion via enhanced pro-IL-1β processing in the cytosol [75], which could explain the lack of rescue observed in the longer duration 3D culture experiments.

The final NF-_K_B inhibitor we tested, PF-06650833, selectively targets IRAK4 and recently attenuated whole-blood interferon gene signatures in humans, collagen-induced arthritis in mice, and inflammatory gene expression in IL-1β-primed human stromal cells when used at 100 nM in vitro [52]. We also observed a rescue in the expression of *FGF19*, *MMP8* and *IL-1β*, when 100 nM of PF-06650833 was co-administered with IL-1β. However, these findings did not translate to 3D culture, as gel contraction and IL6 secretion were similar across conditions. The rescue in *MMP* expression but not cytokine release is consistent with other studies [52] and could reflect differences in early versus late activation of the MyD88 complex, a key upstream activator of IRAK4 and NF-_K_B [50]. Cytokine release may be regulated by rapid induction of IRAK4/1 signalling to NF-_K_B through early MyD88 activation [76], thereby resulting in poor inhibition of IL6 secretion by PF-06650833. Conversely, modulation of *MMP8*, *FGF19* and *IL-1β* expression may be regulated by delayed activation of MyD88 signalling through IRAK4/2 [77], enabling greater inhibition by PF-06650833. Collectively, all NF-_K_B inhibitors failed to rescue the effect of IL-1β in 3D culture and only IRAK4 inhibition modulated gene expression in 2D culture. Differences in IL-1β dosing strategies and tenocyte origin may account for some of these discrepancies. However, unlike 2D monolayer, the 3D microenvironment evokes unique cell-ECM interactions, migration patterns, cytokine, drug, and oxygen gradients as well as mechanotransduction properties which influence cellular responses to external stimuli [56], as demonstrated for cancer therapeutics [55].

IL1Ra is utilised to treat tissue inflammation both in humans [43] and horses [44], with varying degrees of efficiency. Consistent with previous in vitro studies [27], exogenous IL1Ra at concentrations approximately six-fold greater than IL-1β restored collagen gel contraction to control levels. In the present study, IL1Ra attenuated the number of DE genes and reduced NF-_K_B signalling, suggesting this pathway is involved in regulating tenocyte function. Conversely, the 150 DE genes still impacted by IL-1β in the presence of IL1Ra—including *MMP8*, *CSF3* and *IL6*—are unlikely candidates to mediate collagen gel contraction. It is unclear why IL1Ra did not fully rescue global gene expression concomitant with collagen gel contraction. The lack of rescue in *IL-1β* expression by IL1Ra could provide a positive feedback loop sustaining IL-1β signalling via an autocrine mechanism, or the in vitro half-life of IL1Ra reflects in vivo kinetics [43]. Importantly, transcriptome signatures differ from collagen gel contraction rates in equine tenocytes derived at different development stages [53]. Of the 33 genes predicted to regulate tenocyte function, the nucleotide metabolism genes *ENPP1, 2,* and* 5* exhibited the strongest connections in the network analysis, with *ENPP2* upregulated by IL-1β (Log2FC = 3.3), consistent with previous findings [29]. ENPP2 is a secreted protein which generates extracellular lysophosphatidic acid (LPA), impacting cell proliferation, migration and inflammation [78]. Activation of the ENPP2-LPA axis is NF-_K_B dependant [79] and promotes fibrosis in skeletal muscle [80] and liver [81] and regulates immune function in mice [82]. Pharmacological inhibition of ENPP2 signalling to treat pulmonary fibrosis reached phase 3 clinical trials (ClinicalTrials.gov Identifier: NCT03711162), although there are no studies published on tendon. The only ECM-related gene in the network analysis was *AB13BP* which was downregulated by IL-1β (Log2FC = −3.6), consistent with previous studies [83]. Induction of *AB13BP* inhibits tumour growth by promoting senescence [84] and enhances cell-ECM interactions in the vasculature [85]. Based on these observations, we propose that exogenous IL-1β stimulation impairs tenocyte collagen gel contraction through the induction of NF-_K_B-ENPP2-LPA signalling with a simultaneous decrease in ABI3BP-mediated cell-ECM interactions which may impair tenocyte-collagen adhesion. However, transforming growth factor β (TGFβ) has also been shown to promote ENPP2 expression [86] and our data demonstrated a decrease in the expression of genes mapping to enrichment terms involved in TGFβ signalling. Therefore future work to determine the regulation of ENPP2-LPA signaling is required.

There are several limitations to the current study. Firstly, while culturing tenocytes in 3D collagen gels evokes unique transcriptional profiles relative to standard 2D monolayer protocols [53], reconstituted collagen hydrogels fail to fully capture physiological tissue architecture [87]. Furthermore, matrix mechanics and collagen type 1 self-assembly varies depending on the species source and concentration of collagen [88]. Secondly, we investigated the impact of IL-1β on a single cell population, yet multiple cell lineages are present within the tendon niche [67] which likely play a key role in tendon healing [7]. Thirdly, we utilised relatively high passage numbers (P8) in some of the experiments, with studies suggesting phenotypic drift with extended time in culture [89]. However, equine tenocyte gene expression profiles remain stable up to passage 10 [53], suggesting our results still reflect an accurate in vitro model. Fourthly, we did not use vehicle controls, although it is unlikely the diluents (water, PBS, culture media) at the concentrations utilised (1:100–1:1000) would have provoked any meaningful response relative to the unstimulated controls utilised in the current study. Lastly, some of the JSH23 experiments were *n* = 2 (Fig. 3), thus preventing statistical analysis. Both replicates showed good agreement and coupled with the lack of rescue in the 2D qPCR and 3D IL6 secretion measurements, we decided not to take this inhibitor forward to the next stage of experiments.

In conclusion, IL-1β impairs 3D collagen gel contraction and modulates global gene expression profiles through an upregulation of NF-_K_B signalling. Pharmacologically inhibiting the NF-_K_B pathway did not rescue the effects of IL-1β in 3D culture, but the provision of IL1Ra fully restored tenocyte collagen gel contraction and partially rescued global gene expression, including many genes related to NF-_K_B signalling. As IL1Ra is administered daily to reduce inflammation in vivo [43], future work should examine if more frequent administration of the NF-_K_B inhibitors restores collagen gel contraction when co-administered with IL-1β in vitro. Furthermore, inhibiting ENPP2/ABI3BP signalling through genetic and pharmacological approaches should be explored as potential avenues to reduce IL-1β-induced inflammation in adult tenocytes in vitro.

### Supplementary Information

Below is the link to the electronic supplementary material.

Fig. S1 Cytokine and chemokine analysis after 14 days in 3D culture. A Imaged membranes displaying three of ten proteins analysed from the control (top) and IL-1ß (bottom) day 14 spent media samples; the four spots in the top left and two in the bottom right of the membranes depict the positive controls. B Fold change in protein expression for IL8, MCP1, and VEGFα, respectively. *denotes p<0.05 versus control determined by an independent t-test. Values are mean ± SEM of n=3. C The normalised count data from the RNA sequencing analysis for eight of the ten cytokines and chemokines; IL2 and IL4 had zero counts mapped to their respective genes. *denotes p<0.05 versus control based on the DESeq2 analysis (adjusted p value <0.05 and log2-fold change ±1). Values are mean ± SEM of n=5
Supplementary file1 (TIF 657 KB)

Fig. S2 Gene Ontology and Enrichr pathway analysis of the top hits for the upregulated and downregulated DE genes. A Dot plot of the top ten Gene Ontology hits (of 159 enriched terms) for the upregulated genes. B Dot plot of the top ten Gene Ontology hits (of 198 enriched terms) for the downregulated genes. C Bar chart of the top ten Enirchr pathway analysis hits (of 104 terms) for the upregulated genes. D Bar chart of the top ten Enirchr pathway analysis hits (of 31 terms) for the downregulated genes
Supplementary file2 (TIF 729 KB)

Fig. S3 Impact of JSH23 on cell viability in 2D and 3D culture. A Presto blue assay performed in 2D culture following 72 hr stimulation with JSH23 (1, 10, 100 µM) with and without IL-1ß (1 nM). Following a significant one-way ANOVA (p=0.017), pairwise comparisons revealed differences between IL-1ß versus 100 µM JSH23 and IL-1ß versus IL-1ß + 100 µM JSH23 (#p<0.05). Values are mean ± SEM of n=3. B Percentage of cells remaining at day 14 relative to number of cells seeded at day 0 in 3D culture. Values are mean ± SEM of n=2. Light and dark coloured bars represent JSH23-only and JSH23 with IL-1ß conditions, respectively
Supplementary file3 (TIF 472 KB)

Fig. S4 Impact of IMD0354 on cell viability in 2D and 3D culture. A Presto blue assay performed in 2D culture following 72 hr stimulation with IMD0354 (10, 100, 1000 nM) with and without IL-1ß (1 nM). Following a significant one-way ANOVA (p=0.001), pairwise comparisons revealed differences between control versus 1000 nM IMD0354 and control versus IL-1ß + 1000 nM IMD0354 (*p<0.05). B Percentage of cells remaining at day 14 relative to number of cells seeded at day 0 in 3D culture. There was no significant one-way ANOVA (p=0.273). Values are mean ± SEM of n=3. Light and dark coloured bars represent IMD0354-only and IMD0354 with IL-1ß conditions, respectively
Supplementary file4 (TIF 455 KB)

Fig. S5 Impact of PF-06650833 on cell viability in 2D and 3D culture. A Presto blue assay performed in 2D culture following 72 hr stimulation with PF-06650833 (100, 500, 1000 nM) with and without IL-1ß (1 nM). There was no significant one-way ANOVA (p=0.450). B Percentage of cells remaining at day 14 relative to number of cells seeded at day 0 in 3D culture. There was no significant one-way ANOVA (p=0.068). Values are mean ± SEM of n=3. Light and dark coloured bars represent PF-06650833-only and PF-06650833 with IL-1ß conditions, respectively
Supplementary file5 (TIF 450 KB)

Fig. S6 Impact of IL1Ra on 3D gene expression and collagen gel contraction. A Effect of IL1Ra (100 ng/mL) on the expression of ten genes impacted by IL-1ß after 14 days in 3D culture. B Daily collagen gel contraction rates over the 14-day period in the control, IL-1ß (1 nM), IL1Ra (100 ng/mL), and IL-1ß + IL1Ra conditions, respectively. Values are displayed as percentage change relative to day 0. *denotes p<0.05 versus control with a Bonferroni post-hoc following a significant (p<0.05) two-way ANOVA. The IL-1ß + IL1Ra condition was not different from control at any timepoint (p<0.05). Values are mean ± SEM of n=3
Supplementary file6 (TIF 490 KB)

Fig. S7 Functional interaction partners of AB13BP. Network analysis highlighting the top 20 functional interaction partners of AB13B identified in STRING. Proteins (nodes) with a predicted interaction score >0.4 (medium confidence) are displayed. A thicker line (edges) indicates a stronger interaction score. Gene Ontology enrichment analysis revealed significant hits (FDR<0.05) in the cellular component category shown in red (GO:0031012) and blue (GO:0062023), respectively
Supplementary file7 (TIF 685 KB)

Online Resource 1: Animation of the whole-mount confocal images of a day 14 control 3D tenocyte gel. Staining for nuclei, actin, and collagen 1 are shown in blue, green, and red, respectively.
Supplementary file8 (MP4 7241 KB)

Online Resource 2: Animation of the whole-mount confocal images of a day 14 IL-1ß-treated 3D tenocyte gel. Staining for nuclei, actin, and collagen 1 are shown in blue, green, and red, respectively.
Supplementary file9 (MP4 12220 KB)

Online Resource 3: The 954 DE genes upregulated by IL-1ß
Supplementary file10 (CSV 8 KB)

Online Resource 4: The 1563 DE genes downregulated by IL-1ß
Supplementary file11 (CSV 13 KB)

Online Resource 5: The normalised counts for all 29196 genes under control and IL-1ß-treated conditions
Supplementary file12 (CSV 2596 KB)

Online Resource 6: GO-slim enrichment analysis for the 954 DE genes upregulated by IL-1ß
Supplementary file13 (CSV 186 KB)

Online Resource 7: Enrichr pathway enrichment analysis for the 954 DE genes upregulated by IL-1ß
Supplementary file14 (CSV 121 KB)

Online Resource 8: Overlap of DE genes impacted by IL-1ß in the current study vs Gehwolf et al [29]
Supplementary file15 (CSV 16 KB)

Online Resource 9: The 590 DE genes following the control vs IL-1ß pairwise comparison
Supplementary file16 (CSV 44 KB)

Online Resource 10: The 150 DE genes following the control vs IL-1ß + IL1Ra pairwise comparison
Supplementary file17 (CSV 11 KB)

Online Resource 11: The 54 DE genes following the IL-1ß vs IL-1ß + IL1Ra pairwise comparison
Supplementary file18 (CSV 4 KB)

Online Resource 12: GO-slim enrichment analysis for the 590 DE genes comparing IL-1ß vs control reveals enrichment for NF-KB signalling
Supplementary file19 (CSV 11 KB)

Online Resource 13: GO-slim enrichment analysis for the 150 DE genes comparing IL-1ß vs IL-ß + IL1Ra reveals no enrichment for NF-KB signalling
Supplementary file20 (CSV 5 KB)

Online Resource 14: The 33 DE genes suggested to mediate tenocyte-induced collagen gel contraction
Supplementary file21 (CSV 0 KB)

## Data Availability

All relevant data are within the manuscript and the supplementary figures. The RNA sequencing data are available through NCBI GEO (https://www.ncbi.nlm.nih.gov/geo/) under accession number GSE221370.
