# Supplementary figures and images for: Exogenous interleukin-1 beta stimulation regulates equine tenocyte function and gene expression in three-dimensional culture which can be rescued by pharmacological inhibition of interleukin 1 receptor, but not nuclear factor kappa B, signaling
Source: Mol Cell Biochem. 2023 Jun 14;479(5):1059–78. doi: 10.1007/s11010-023-04779-z (PMC11116237; doi:10.1007/s11010-023-04779-z)

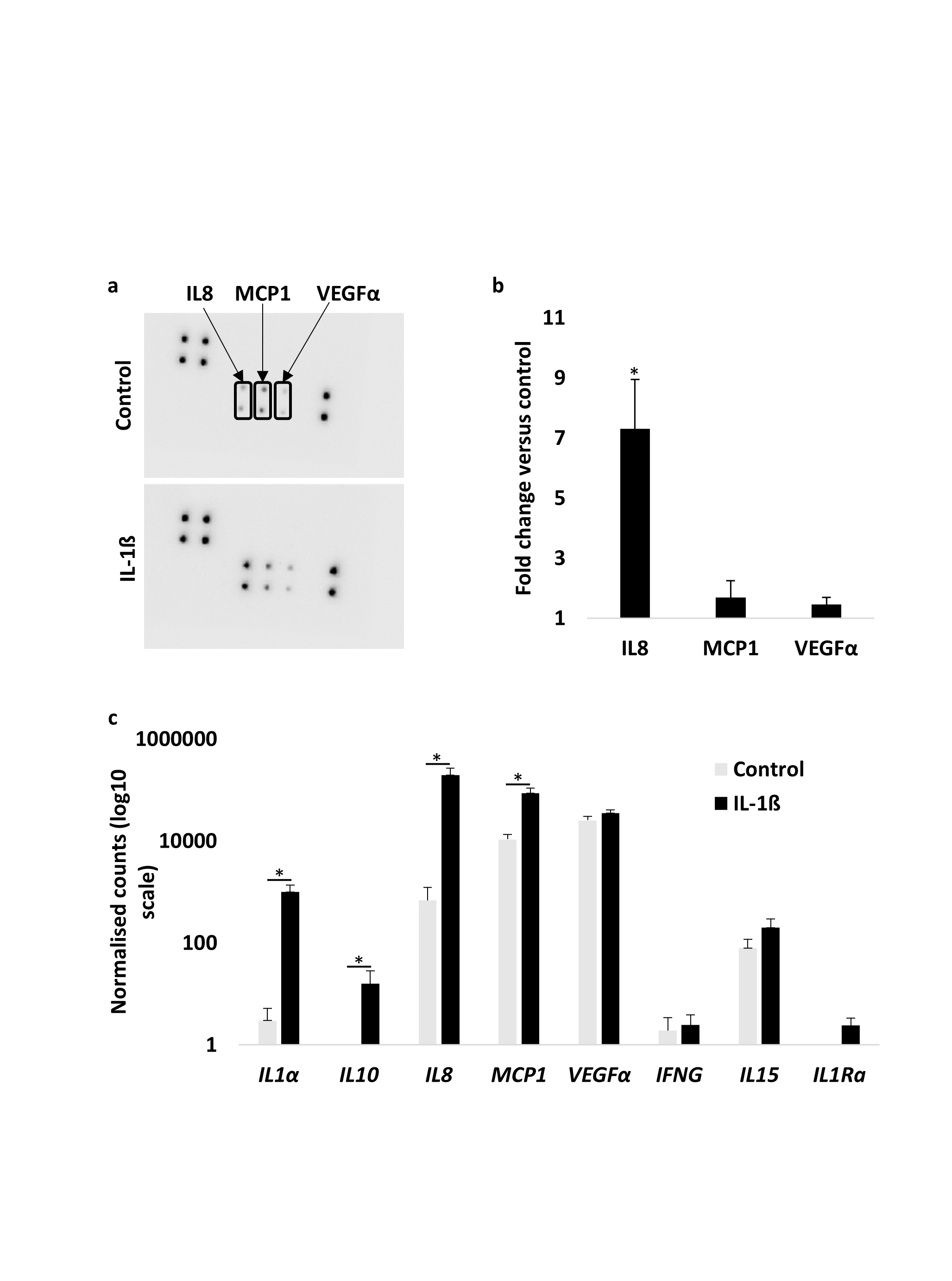

Supplement: Supplementary file 1 — Fig. S1 Cytokine and chemokine analysis after 14 days in 3D culture. A Imaged membranes displaying three of ten proteins analysed from the control (top) and IL-1ß (bottom) day 14 spent media samples; the four spots in the top left and two in the bottom right of the membranes depict the positive controls. B Fold change in protein expression for IL8, MCP1, and VEGFα, respectively. *denotes p<0.05 versus control determined by an independent t-test. Values are mean ± SEM of n=3. C The normalised count data from the RNA sequencing analysis for eight of the ten cytokines and chemokines; IL2 and IL4 had zero counts mapped to their respective genes. *denotes p<0.05 versus control based on the DESeq2 analysis (adjusted p value <0.05 and log2-fold change ±1). Values are mean ± SEM of n=5 Supplementary file1 (TIF 657 KB) [file 11010_2023_4779_MOESM1_ESM.tif]

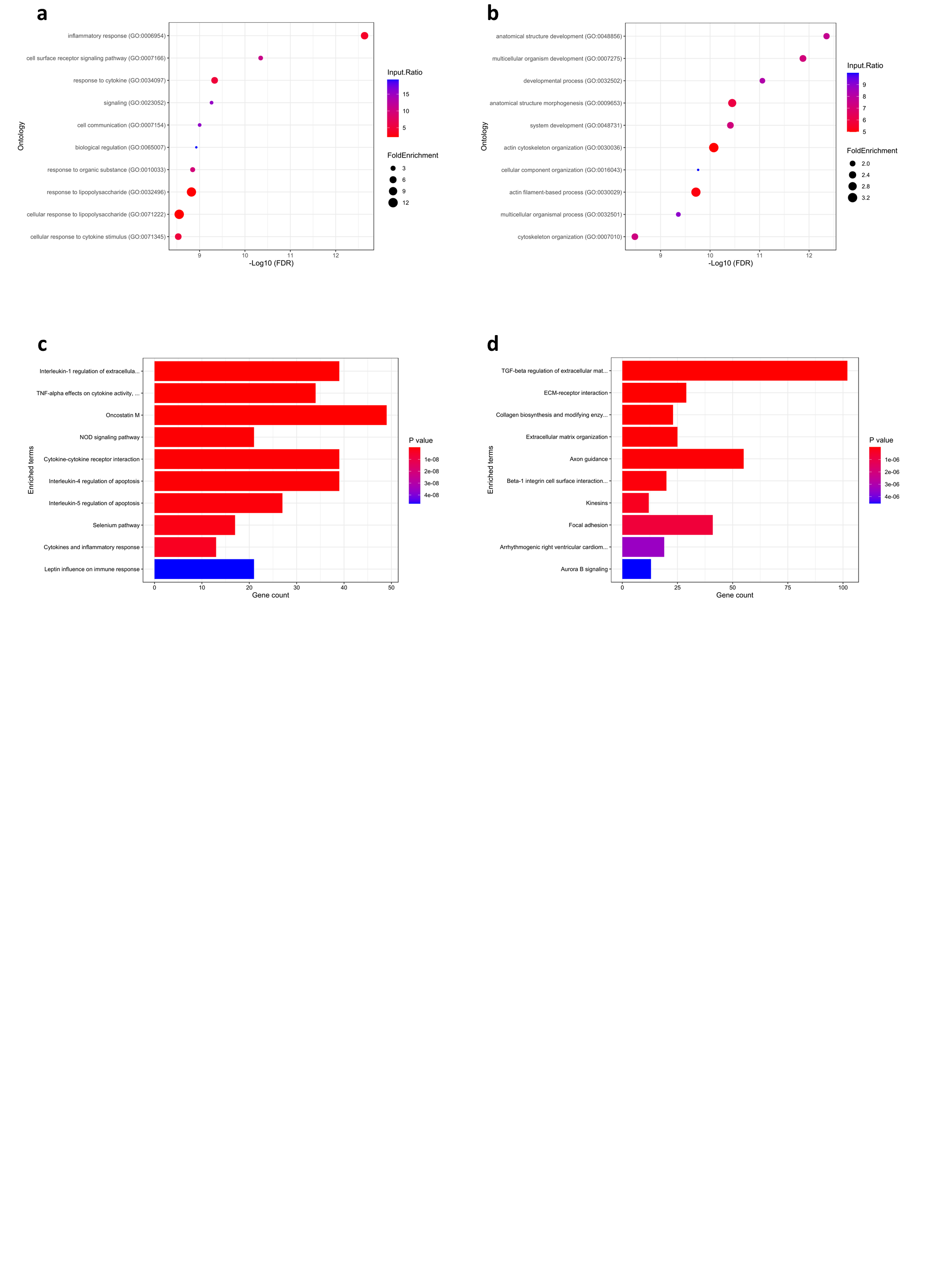

Supplement: Supplementary file 2 — Fig. S2 Gene Ontology and Enrichr pathway analysis of the top hits for the upregulated and downregulated DE genes. A Dot plot of the top ten Gene Ontology hits (of 159 enriched terms) for the upregulated genes. B Dot plot of the top ten Gene Ontology hits (of 198 enriched terms) for the downregulated genes. C Bar chart of the top ten Enirchr pathway analysis hits (of 104 terms) for the upregulated genes. D Bar chart of the top ten Enirchr pathway analysis hits (of 31 terms) for the downregulated genes Supplementary file2 (TIF 729 KB) [file 11010_2023_4779_MOESM2_ESM.tif]

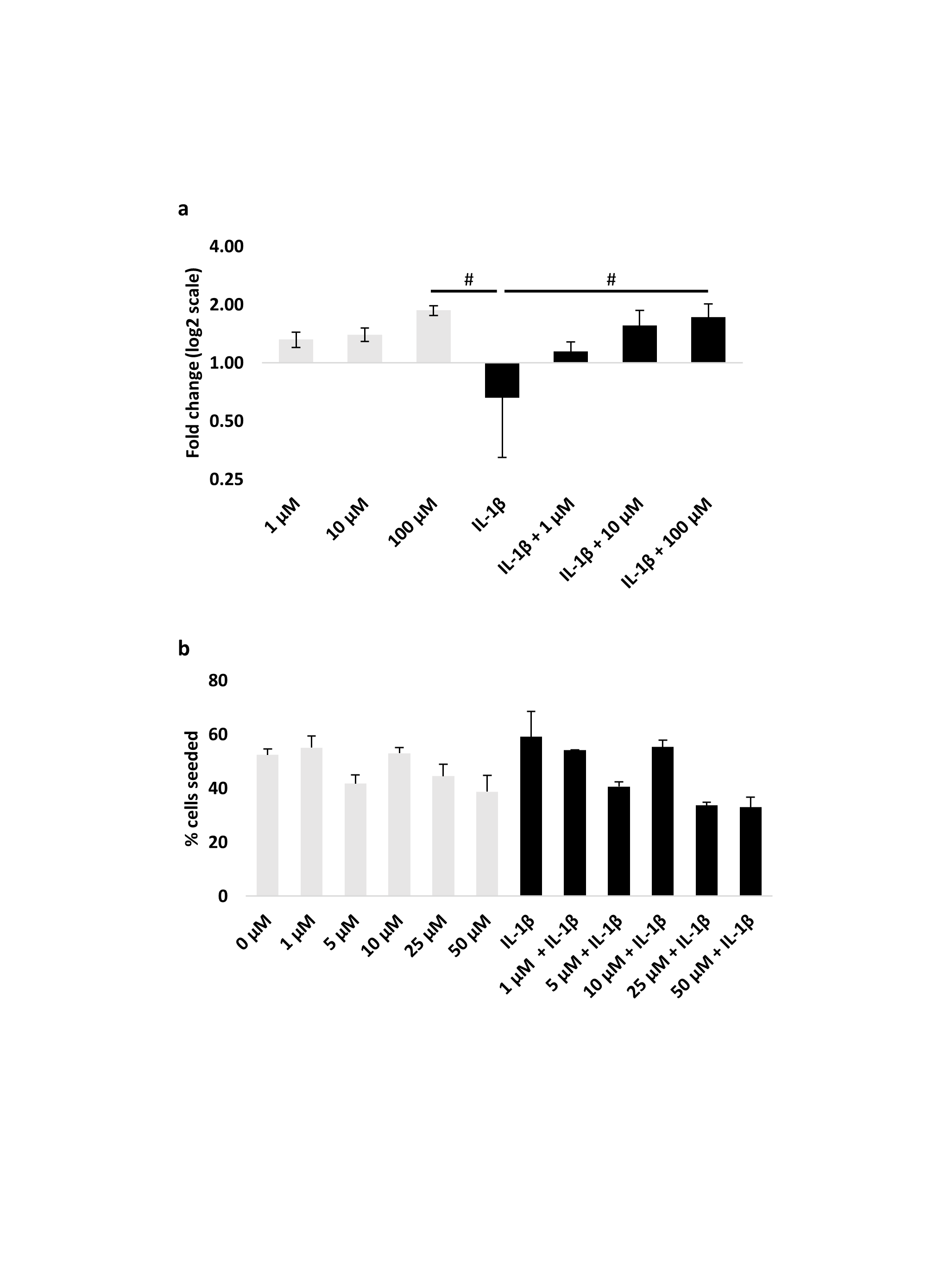

Supplement: Supplementary file 3 — Fig. S3 Impact of JSH23 on cell viability in 2D and 3D culture. A Presto blue assay performed in 2D culture following 72 hr stimulation with JSH23 (1, 10, 100 µM) with and without IL-1ß (1 nM). Following a significant one-way ANOVA (p=0.017), pairwise comparisons revealed differences between IL-1ß versus 100 µM JSH23 and IL-1ß versus IL-1ß + 100 µM JSH23 (#p<0.05). Values are mean ± SEM of n=3. B Percentage of cells remaining at day 14 relative to number of cells seeded at day 0 in 3D culture. Values are mean ± SEM of n=2. Light and dark coloured bars represent JSH23-only and JSH23 with IL-1ß conditions, respectively Supplementary file3 (TIF 472 KB) [file 11010_2023_4779_MOESM3_ESM.tif]

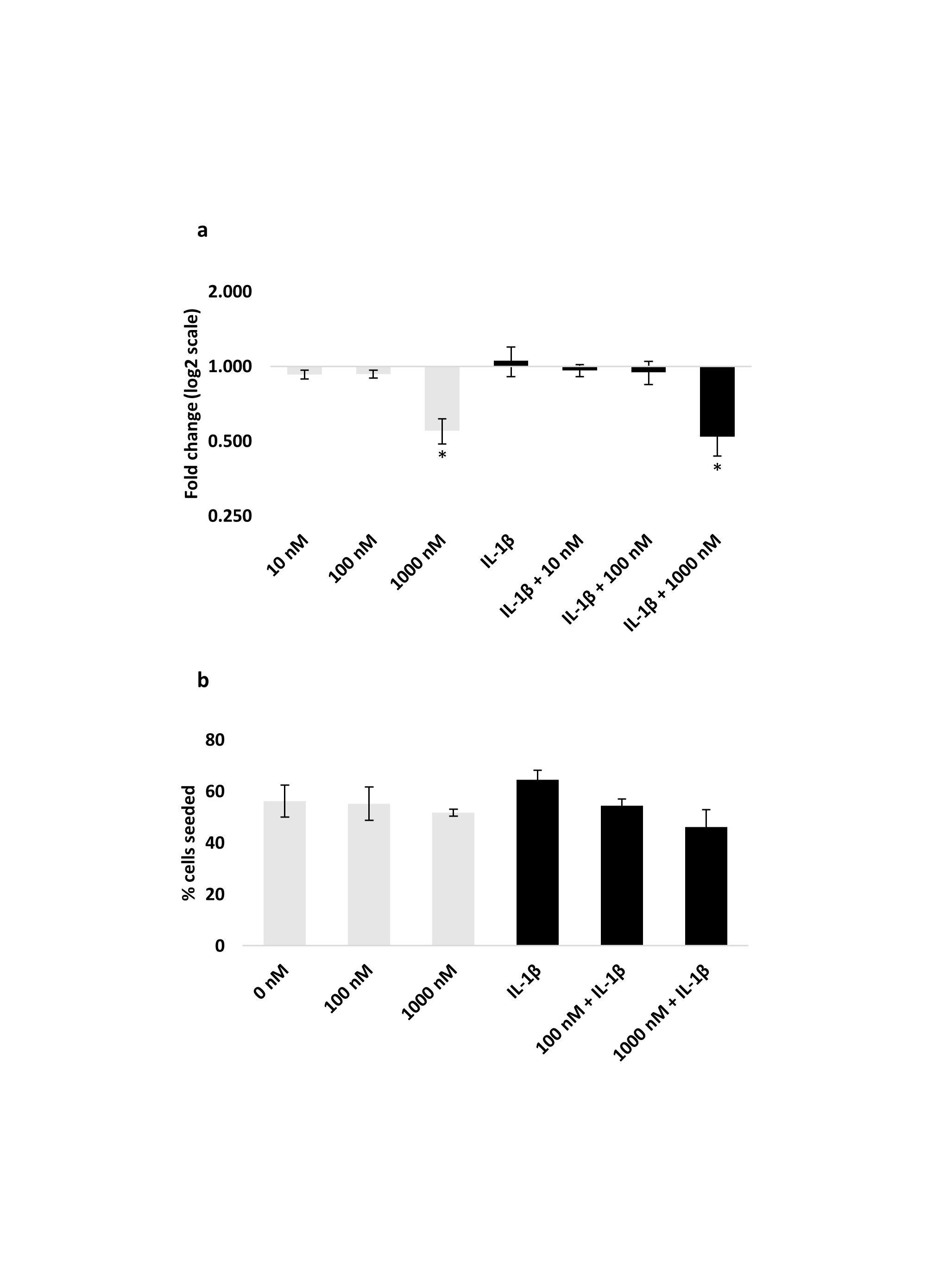

Supplement: Supplementary file 4 — Fig. S4 Impact of IMD0354 on cell viability in 2D and 3D culture. A Presto blue assay performed in 2D culture following 72 hr stimulation with IMD0354 (10, 100, 1000 nM) with and without IL-1ß (1 nM). Following a significant one-way ANOVA (p=0.001), pairwise comparisons revealed differences between control versus 1000 nM IMD0354 and control versus IL-1ß + 1000 nM IMD0354 (*p<0.05). B Percentage of cells remaining at day 14 relative to number of cells seeded at day 0 in 3D culture. There was no significant one-way ANOVA (p=0.273). Values are mean ± SEM of n=3. Light and dark coloured bars represent IMD0354-only and IMD0354 with IL-1ß conditions, respectively Supplementary file4 (TIF 455 KB) [file 11010_2023_4779_MOESM4_ESM.tif]

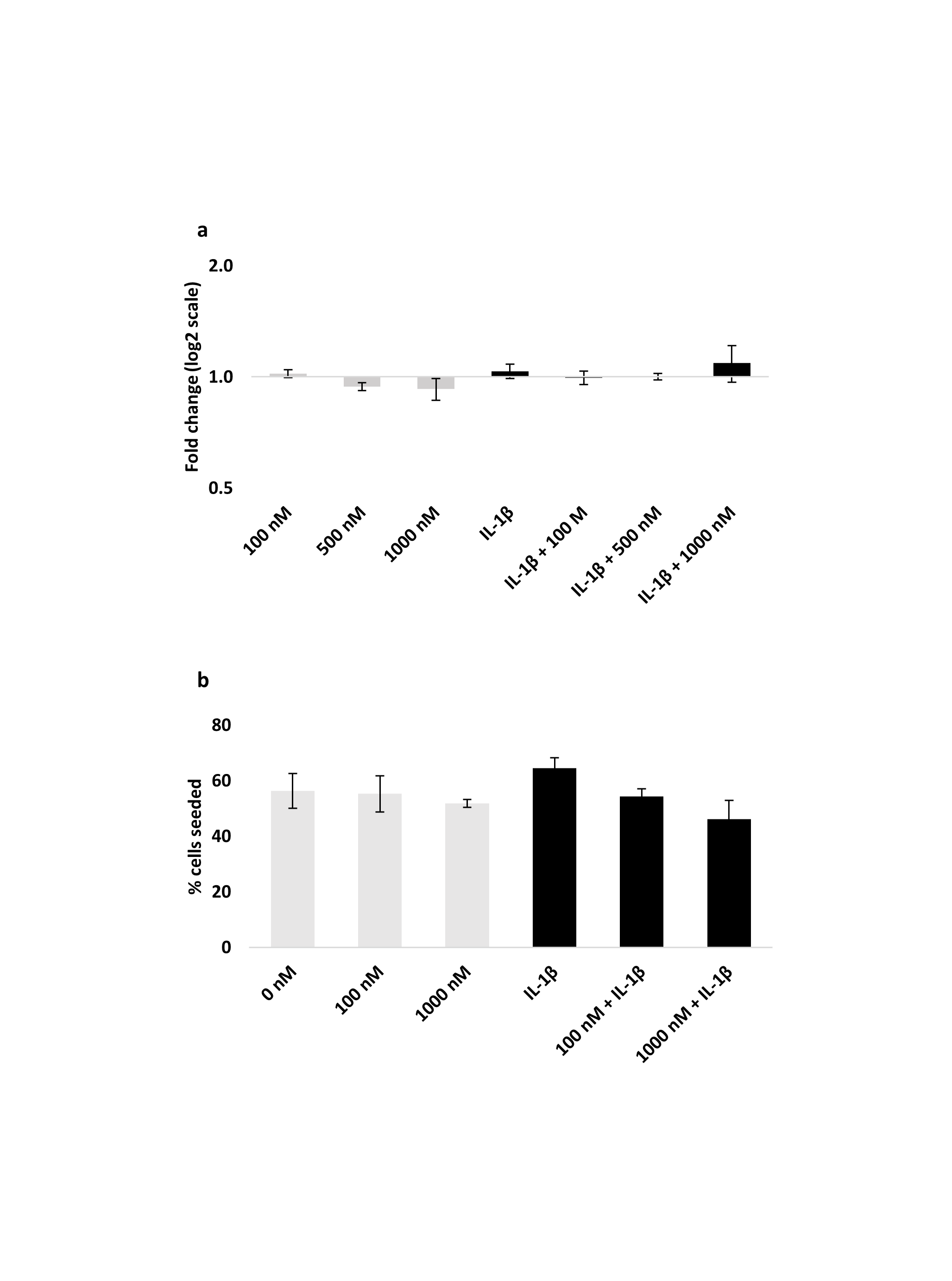

Supplement: Supplementary file 5 — Fig. S5 Impact of PF-06650833 on cell viability in 2D and 3D culture. A Presto blue assay performed in 2D culture following 72 hr stimulation with PF-06650833 (100, 500, 1000 nM) with and without IL-1ß (1 nM). There was no significant one-way ANOVA (p=0.450). B Percentage of cells remaining at day 14 relative to number of cells seeded at day 0 in 3D culture. There was no significant one-way ANOVA (p=0.068). Values are mean ± SEM of n=3. Light and dark coloured bars represent PF-06650833-only and PF-06650833 with IL-1ß conditions, respectively Supplementary file5 (TIF 450 KB) [file 11010_2023_4779_MOESM5_ESM.tif]

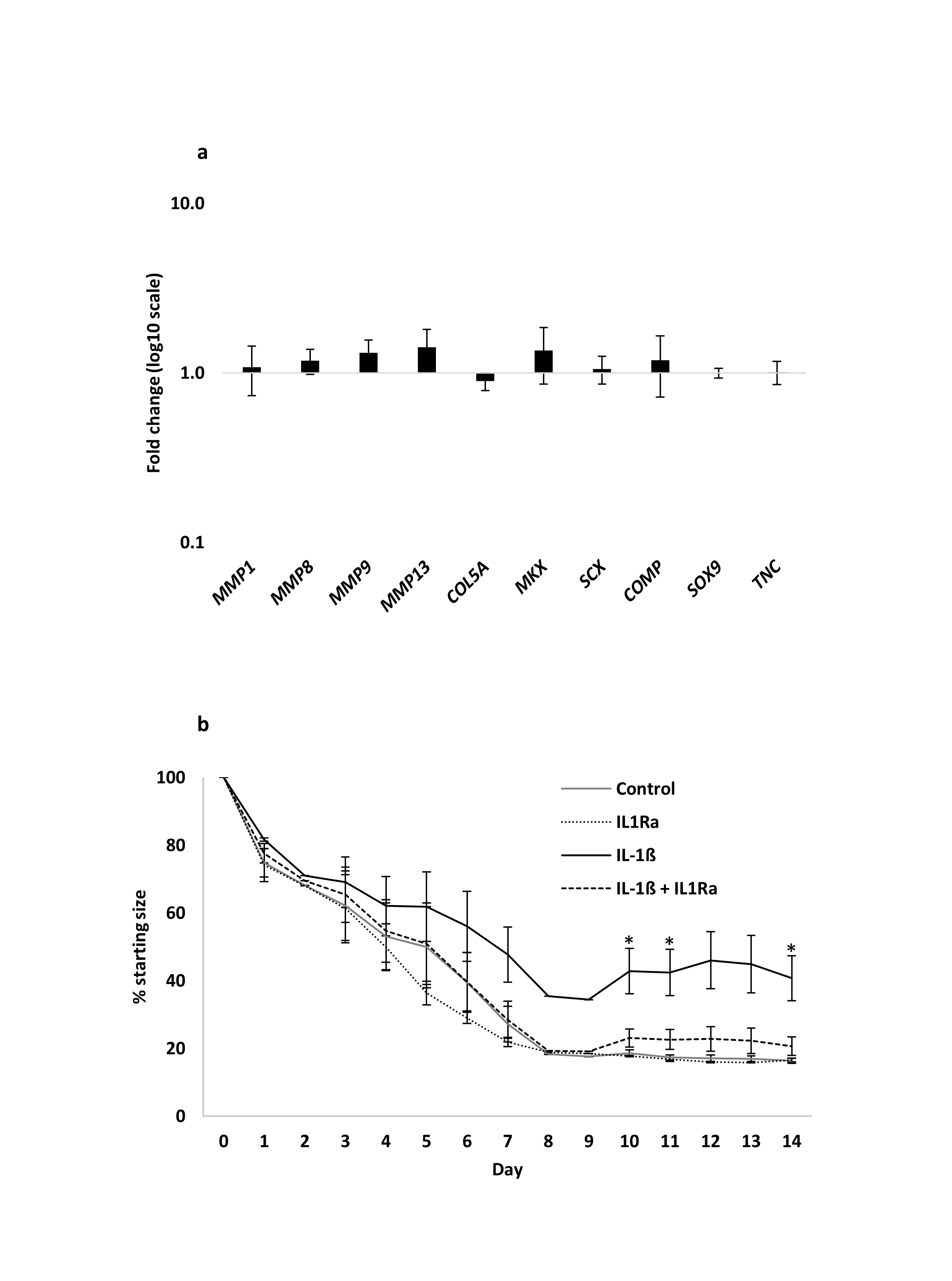

Supplement: Supplementary file 6 — Fig. S6 Impact of IL1Ra on 3D gene expression and collagen gel contraction. A Effect of IL1Ra (100 ng/mL) on the expression of ten genes impacted by IL-1ß after 14 days in 3D culture. B Daily collagen gel contraction rates over the 14-day period in the control, IL-1ß (1 nM), IL1Ra (100 ng/mL), and IL-1ß + IL1Ra conditions, respectively. Values are displayed as percentage change relative to day 0. *denotes p<0.05 versus control with a Bonferroni post-hoc following a significant (p<0.05) two-way ANOVA. The IL-1ß + IL1Ra condition was not different from control at any timepoint (p<0.05). Values are mean ± SEM of n=3 Supplementary file6 (TIF 490 KB) [file 11010_2023_4779_MOESM6_ESM.tif]

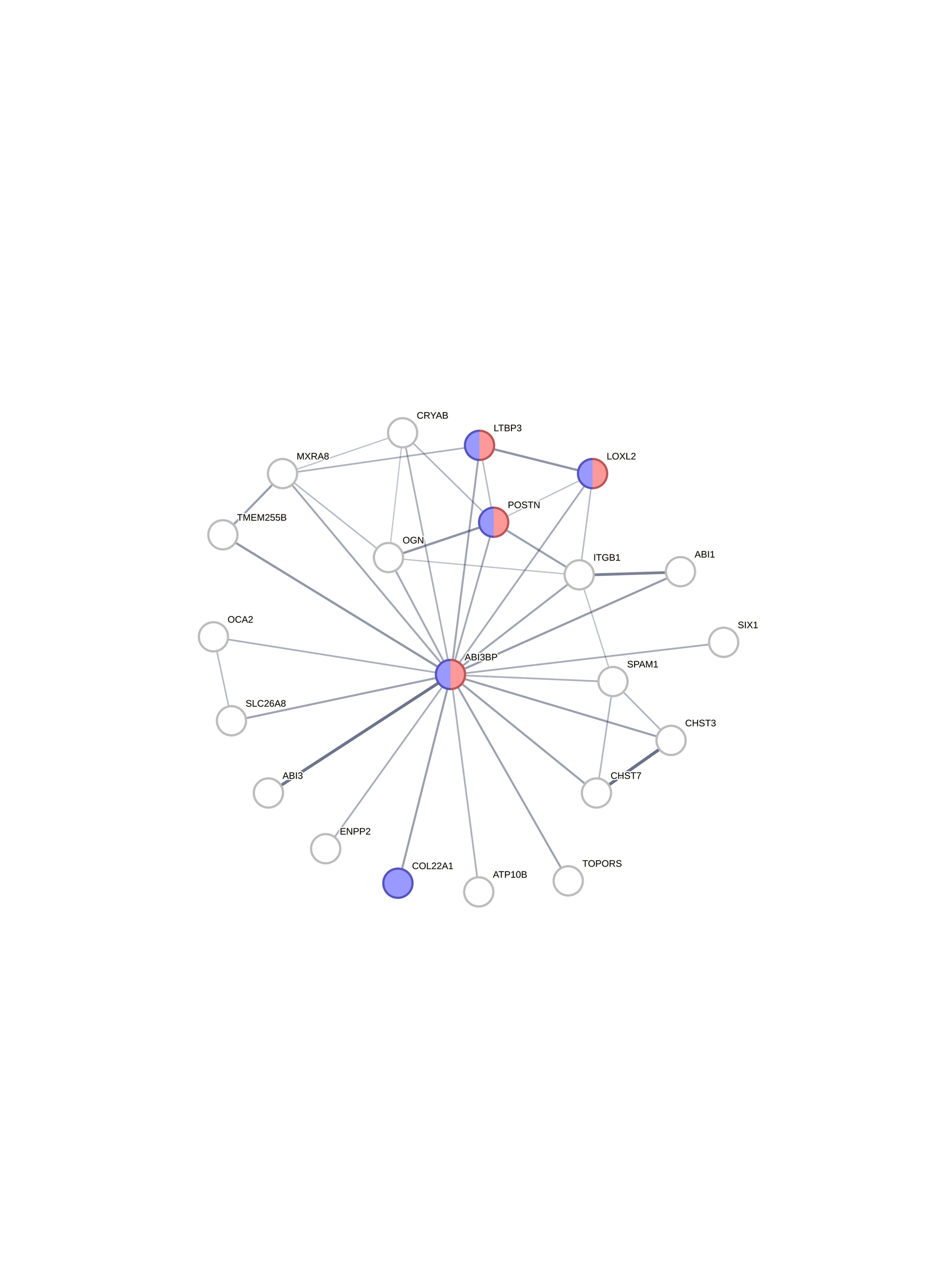

Supplement: Supplementary file 7 — Fig. S7 Functional interaction partners of AB13BP. Network analysis highlighting the top 20 functional interaction partners of AB13B identified in STRING. Proteins (nodes) with a predicted interaction score >0.4 (medium confidence) are displayed. A thicker line (edges) indicates a stronger interaction score. Gene Ontology enrichment analysis revealed significant hits (FDR<0.05) in the cellular component category shown in red (GO:0031012) and blue (GO:0062023), respectively Supplementary file7 (TIF 685 KB) [file 11010_2023_4779_MOESM7_ESM.tif]
